# Long Noncoding RNAs and Circular RNAs Regulate AKT and Its Effectors to Control Cell Functions of Cancer Cells

**DOI:** 10.3390/cells11192940

**Published:** 2022-09-20

**Authors:** Jen-Yang Tang, Ya-Ting Chuang, Jun-Ping Shiau, Kun-Han Yang, Fang-Rong Chang, Ming-Feng Hou, Ammad Ahmad Farooqi, Hsueh-Wei Chang

**Affiliations:** 1 School of Post-Baccalaureate Medicine, Kaohsiung Medical University, Kaohsiung 80708, Taiwan; 2 Department of Radiation Oncology, Kaohsiung Medical University Hospital, Kaoshiung Medical University, Kaohsiung 80708, Taiwan; 3 Graduate Institute of Medicine, College of Medicine, Kaohsiung Medical University, Kaohsiung 80708, Taiwan; 4 Division of Breast Oncology and Surgery, Department of Surgery, Kaohsiung Medical University Hospital, Kaohsiung Medical University, Kaohsiung 80708, Taiwan; 5 Graduate Institute of Natural Products, Kaohsiung Medical University, Kaohsiung 80708, Taiwan; 6 Department of Biomedical Science and Environmental Biology, College of Life Science, Kaohsiung Medical University, Kaohsiung 80708, Taiwan; 7 Institute of Biomedical and Genetic Engineering (IBGE), Islamabad 54000, Pakistan; 8 Center for Cancer Research, Kaohsiung Medical University, Kaohsiung 80708, Taiwan

**Keywords:** lncRNA, circRNA, AKT, cell functions, cancer

## Abstract

AKT serine-threonine kinase (AKT) and its effectors are essential for maintaining cell proliferation, apoptosis, autophagy, endoplasmic reticulum (ER) stress, mitochondrial morphogenesis (fission/fusion), ferroptosis, necroptosis, DNA damage response (damage and repair), senescence, and migration of cancer cells. Several lncRNAs and circRNAs also regulate the expression of these functions by numerous pathways. However, the impact on cell functions by lncRNAs and circRNAs regulating AKT and its effectors is poorly understood. This review provides comprehensive information about the relationship of lncRNAs and circRNAs with AKT on the cell functions of cancer cells. the roles of several lncRNAs and circRNAs acting on AKT effectors, such as FOXO, mTORC1/2, S6K1/2, 4EBP1, SREBP, and HIF are explored. To further validate the relationship between AKT, AKT effectors, lncRNAs, and circRNAs, more predicted AKT- and AKT effector-targeting lncRNAs and circRNAs were retrieved from the LncTarD and circBase databases. Consistently, using an in-depth literature survey, these AKT- and AKT effector-targeting database lncRNAs and circRNAs were related to cell functions. Therefore, some lncRNAs and circRNAs can regulate several cell functions through modulating AKT and AKT effectors. This review provides insights into a comprehensive network of AKT and AKT effectors connecting to lncRNAs and circRNAs in the regulation of cancer cell functions.

## 1. Introduction

**Figure 1:**
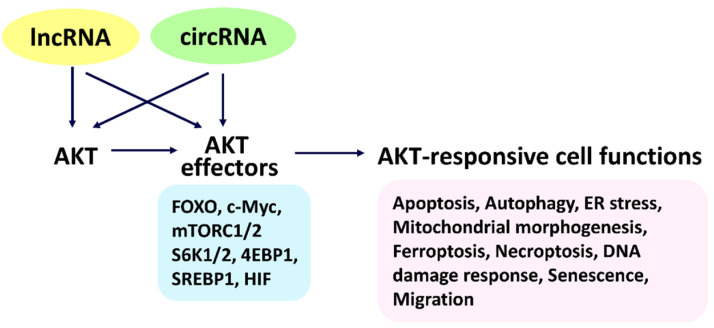
Overview of AKT, AKT effectors, lncRNAs, and circRNAs regulating diverse cancer cell functions.

AKT serine/threonine kinase (AKT) shows activation or overexpression in several cancers [1]. AKT signaling is vital for diverse regulations to modulate several cell functions [2,3,4], such as survival, proliferation, metabolism, and angiogenesis. Additionally, several AKT signaling-associated cell functions include apoptosis, autophagy, endoplasmic reticulum (ER) stress, mitochondrial morphogenesis (fission/fusion), ferroptosis, necroptosis, and DNA damage response (damage and repair), senescence, and migration (Figure 1). AKT mutation occurs in several cancer types, such as leukemia [5], breast [6], and others [7]. However, AKT mutation rates seem low (3–5%) for all cancers [7,8], and this issue was not included in this review.

AKT signaling can modulate several downstream AKT effectors, such as forkhead box transcription factors (FOXO), c-Myc, mechanistic target of rapamycin (mTOR) complex 1/2 (mTORC1/2), mTOR substrate S6 kinase 1/2 (S6K1/2; RPS6KB1/2), eukaryotic translation initiation factor 4E-binding protein 1 (4EBP1; EIF4EBP1), sterol regulatory element-binding protein 1 (SREBP1; SREBF1), and hypoxia-inducible factor (HIF) [3,9,10,11] (Figure 1). However, the complex functions of AKT effects on these cell functions need further investigation.

Emerging evidence has shown the impacts of noncoding RNA (ncRNAs), such as long noncoding RNAs (lncRNAs) and circular RNAs (circRNAs), on regulating cell progression, especially relevant in cancer cells [12]. LncRNAs are a group of ncRNA molecules containing more than 200 nucleotides. LncRNAs exhibit complex interactions with microRNAs (miRNAs), mRNAs, and proteins to regulate cell functions [13]. LncRNAs are essential modulators for regulating gene expression and affect diverse cell functions [14]. CircRNAs are other ncRNAs formed by splicing and sequentially connecting between splice donor and acceptor sites, i.e., backsplicing [15]. CircRNAs may function as protein and RNA scaffolds to bind miRNA and regulate transcription or translation in a miRNA and RNA-binding protein sponge manner [16,17].

Both lncRNAs [18,19,20] and circRNAs [21,22] are reported as potential tumor markers by regulating numerous pathways and controlling cell functions. Mounting evidence shows the interaction between ncRNAs and AKT signaling in several cancers [23,24,25]. As mentioned above, this review focuses on understanding the relationship between AKT and AKT effectors in lncRNA- and circRNA-modulating cell functions. However, the network between AKT, AKT signaling, lncRNAs, and circRNAs lacks systemic integration. In previous reports, possible cell functions were not connected to this AKT–AKT effectors–lncRNAs–circRNAs network. This warrants a detailed organization for understanding their relationships.

LncRNAs [26] and circRNAs [27] can function as miRNA sponges, modulating their target mRNA expressions. However, the miRNA information was not under the scope of this review and is not discussed. Additionally, lncRNAs can interact with DNA, RNA, and proteins for gene regulation [28]. Several DNA and proteins targeted by lncRNAs were summarized in regulating transcription, posttranscription, cellular organelles, structural functions, and genomic integrity [28]. For example, lncRNAs can control chromatin regulation through the recruitment of chromatin modifiers, decoy of chromatin modifiers, and the direct cis or trans interaction with chromatin [28]. LncRNAs also control transcription regulation through target-gene inhibition, gene activation, and multiple lncRNAs acting on the same locus. Consequently, the detailed mechanisms for lncRNAs and circRNAs to control gene expressions are complex and display distinct regulations in different cases. Therefore, the complicated mechanisms of the interactions between AKT/AKT effectors and their respective lncRNAs and circRNAs are not included in this review. Alternatively, we focused on potential target genes such as AKT and AKT effectors regulated by lncRNAs and circRNAs that were predicted by databases, as mentioned later.

This review provides an overview of AKT, AKT effectors, lncRNAs, and circRNAs in regulating cell functions (Figure 1). Firstly, it summarizes detailed information on AKT and AKT signaling-modulated functions relating lncRNAs and circRNAs to several cell functions, especially for cancer cells, including apoptosis, autophagy, ER stress, mitochondrial morphogenesis, ferroptosis, necroptosis, DNA damage response, senescence, and migration. Detailed mechanisms for most lncRNA- and circRNA-associated regulation of AKT and AKT effectors lack in-depth connection. To fill the gap, we next chose databases for lncRNAs and circRNAs that provide the predicted targets to AKT and AKT effectors (Section 2 and Section 3). Subsequently, these predicted targets (AKT and AKT effectors) of lncRNAs and circRNAs are connected to cell functions by a literature survey. Finally, AKT and AKT effectors that regulate cell functions appear to be well organized and connected to lncRNAs and circRNAs.

## 2. Connecting AKT/AKT Effectors and LncRNAs to Cell Functions

The human AKT family contains AKT1, AKT2, and AKT3 [29,30,31], located at chromosomes 14, 19, and 1. These AKT family members share several conserved structures containing the pleckstrin homology (PH) domain at the N-terminal, kinase domain at the middle region, and the hydrophobic regulating domain at C-terminal [32]. AKT1 expresses in ubiquitous tissues, while AKT2 and AKT3 are mainly expressed in skeletal muscle and liver [33] and in brain and testis [34], respectively.

Notably, some lncRNAs were reported to modulate the expressions of AKT1 [35], AKT2 [36], and AKT3 [37]. However, their connection to cell function was not investigated, especially for cancer cells. Hence, the relationship that connects AKT and lncRNAs to their modulating cell functions (Figure 1) was evaluated by literature retrieval (Section 2.1). However, their potential mechanisms still warrant a detailed exploration, particularly for the possible targeting to AKT by lncRNAs. Subsequently, the potential targeting to AKT1, AKT2, and AKT3 by lncRNAs and their associated cell functions are discussed (Section 2.2). By choosing the lncRNA database (LncTarD [38]), the target information of respective lncRNAs was predicted, and their impacts on cell functions were evaluated, as described later.

Some lncRNAs also were reported to modulate the expressions of AKT effectors [38]. However, their connection to cell functions has never been investigated, especially for cancer cells. Hence, the evidence that connects the AKT effectors and lncRNAs to their modulating cell functions (Figure 1) was evaluated by literature retrieval (Section 2.3). However, their potential mechanisms still warrant a detailed assessment, particularly for the possible targeting to AKT effectors by lncRNAs. Subsequently, the potential targeting to AKT effectors by lncRNAs and their associated cell functions are discussed (Section 2.4). By choosing the lncRNA database LncTarD [38], the target information of respective lncRNAs was predicted and their impacts on cell functions evaluated as described later.

### 2.1. Connecting AKT and LncRNAs to Cell Functions

Phosphoinositide 3-kinase (PI3K)/AKT/mTOR signaling and lncRNAs have a cross-relationship regulating carcinogenesis [24,39,40]. They are essential in regulating apoptosis, autophagy, ER stress, mitochondrial morphogenesis, ferroptosis, necroptosis, DNA damage response, senescence, and migration. These AKT-lncRNA--regulating cell functions are discussed in Section 2.1.1, Section 2.1.2, Section 2.1.3, Section 2.1.4, Section 2.1.5, Section 2.1.6 and Section 2.1.7, especially for cancer cells.

#### 2.1.1. Apoptosis by AKT-Regulating LncRNAs

Apoptosis-modulating effects of lncRNAs involving AKT have been reported. Some lncRNA studies reported apoptosis-promoting effects in various cancer cell types connecting to AKT. Phosphatase and tension homolog deleted on chromosome ten (PTEN) is a negative modulator of AKT signaling [41]. LncRNA FER1L4 causes apoptosis of lung cancer cells by upregulating PTEN expression and dephosphorylating AKT [42]. LncRNA LINC00619 is downregulated in osteosarcoma cells, associated with AKT mRNA overexpression and its protein phosphorylation [43]. LINC00619 overexpression promotes apoptosis of osteosarcoma cells by targeting hepatocyte growth factor (HGF) and downregulating AKT mRNA expressions and its protein phosphorylation [43]. Consequently, various lncRNAs may be overexpressed in several cancers to modulate AKT for controlling apoptosis induction.

In contrast, some lncRNA studies reported apoptosis-suppressing effects of several cancer cell types connecting to AKT. LncRNA HOTAIR inhibits apoptosis of retinoblastoma cells by upregulating ribonucleotide reductase regulatory subunit M2 (RRM2) for phosphorylating AKT, reverted by HOTAIR knockdown [44]. LncRNA RP11-301G19.1 downregulation triggers apoptosis of myeloma cancer cells by dephosphorylating AKT [45]. SNHG20 silencing triggers apoptosis of lung cancer cells by dephosphorylating AKT [46]. ROR1-AS1 is overexpressed in lung cancer tissues. ROR1-AS1 inhibition triggers apoptosis in lung cancer cells by dephosphorylating AKT [47]. LINC01410 and lncRNA PITPNA-AS1 are overexpressed in glioblastoma cells [48,49]. LINC01410 knockdown induces temozolomide-induced apoptosis of glioblastoma cells by upregulating PTEN expression and dephosphorylating AKT [48]. LncRNA PITPNA-AS1 inhibits apoptosis of glioblastoma cells by upregulating epidermal growth factor receptor (EGFR) expression and phosphorylating AKT [49].

Accordingly, different lncRNAs may promote or suppress apoptosis by phosphorylating or dephosphorylating AKT to regulate its activity. As mentioned above, some tumors are overexpressed or downregulated by AKT-regulating lncRNAs. A strategy to overexpress or downregulate these specific AKT-regulating lncRNAs can improve the apoptosis-inducible effects for cancer treatment.

#### 2.1.2. Autophagy by AKT-Regulating LncRNAs

Autophagy modulating effects of lncRNAs involving AKT have been reported. The lncRNA MEG3 overexpression blocks the phosphorylation of PI3K/AKT/mTOR to promote autophagy in tumor necrosis factor α (TNF-α)-treated keratinocytes [50]. Some lncRNAs exhibit bifunctional effects to regulate apoptosis and autophagy. LncRNA ADAMTS9-AS1 upregulation blocks apoptosis and autophagy of bladder cancer cells by phosphorylating AKT, reverted by downregulating ADAMTS9-AS1 [51]. Accordingly, different lncRNAs may promote or suppress autophagy by phosphorylating or dephosphorylating AKT to regulate its activity.

#### 2.1.3. ER Stress by AKT-Regulating LncRNAs

Drug-induced ER stress effects of lncRNA involving AKT have been reported. 5-Fluorouracil induces ER stress and glucose-regulated protein 78 (GRP78; BiP) expression of breast cancer cells to cause 5-fluorouracil resistance, accompanied by upregulating myocardial infarction-associated transcript (MIAT lncRNA) and AKT protein expression [52]. This warrants surveying more lncRNAs that regulate other ER stress components in the future.

#### 2.1.4. DNA Damage Response by AKT-Regulating LncRNAs

DNA repair-suppressing effects of lncRNAs involving AKT have been reported. Linc00312 directly targets the DNA-dependent protein kinase, catalytic subunit (DNA-PKcs), blocks the interaction between DNA-PKcs and Ku80, and inactivates AKT by dephosphorylation, suppressing nonhomologous end joining (NHEJ) repair in nasopharyngeal cancer cells [53]. This warrants surveying more lncRNAs that regulate other DNA damage and repair in the future.

#### 2.1.5. Senescence by AKT-Regulating LncRNAs

Senescence-inducible effects of lncRNAs involving AKT have been reported. GAS5 silencing induces proliferation and suppresses the senescence of endothelial progenitor cells by dephosphorylating PI3K/AKT [54]. This warrants a detailed survey and examination of senescence effects of AKT-targeting lncRNAs on cancer cells in the future.

#### 2.1.6. Migration by AKT-Regulating LncRNAs

Drug-induced migration-modulating effects of lncRNAs involving AKT have been reported. Some lncRNA studies investigated migration-promoting effects. PYCR2 knockdown suppresses the migration of colon cancer cells by downregulating matrix metalloproteinase (MMP) 2/9 and dephosphorylating AKT [55]. LINC00963 promotes metastasis of lung cancer cells by phosphorylating AKT [56]. SOX2 overlapping transcript (SOX2-OT) promotes the phosphorylation of PI3K/AKT and induces breast cancer cell metastasis [57]. MIR205HG [58] and AC099850.3 [59] enable the migration of liver cancer cells by phosphorylating AKT.

In contrast, some lncRNA studies investigated migration-suppressing effects. Platelet-derived growth factor BB (PDGF-BB) inhibits RP5-857K21.7 expression of airway smooth muscle cells (ASMCs). RP5-857K21.7 overexpression inhibits the migration of PDGF-BB-treated ASMCs through dephosphorylating AKT [60]. Accordingly, different lncRNAs may promote or suppress migration by phosphorylating or dephosphorylating AKT. This warrants a detailed survey and examination of migration effects of AKT-targeting lncRNAs on cancer cells in the future.

#### 2.1.7. Potential Future Directions

As described above, several lncRNAs were mentioned to regulate AKT phosphorylation or dephosphorylation for its activation and inactivation and, in turn, control cell functions. Some AKT-regulating lncRNAs are abundant or scarce in various cancers. Overexpressing or downregulating these AKT-regulating lncRNAs may reverse the status of cancer cell functions to improve anticancer effects.

However, the cell function mechanism for the modulating effects of lncRNAs on AKT remains unclear, particularly for assessing the potential targeting to AKT by lncRNAs. More experiments are warranted to improve the connection between AKT-lncRNAs regulating cancer cell functions.

### 2.2. Connecting AKT1/AKT2/AKT3 and Database-LncRNAs to Cell Functions

**Figure 2:**
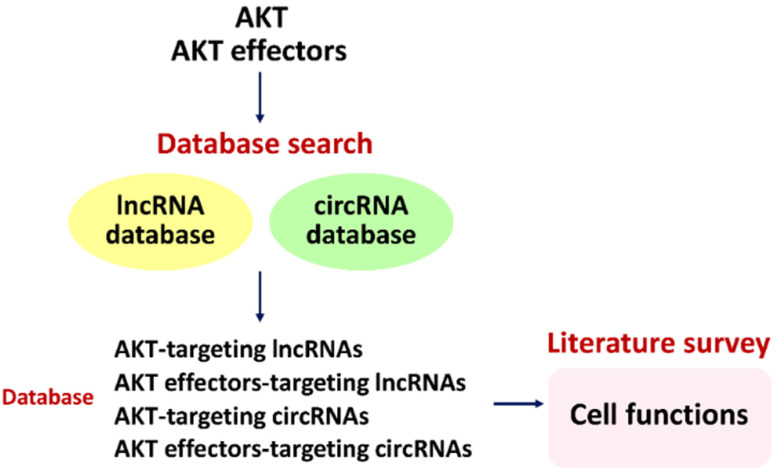
Strategy for connecting database-predicted AKT-, AKT effector-targeting lncRNAs and circRNAs to their regulating cell functions. By searching LncTarD [38] and circBase [61], these AKT- and AKT effector-targeting lncRNA and circRNA candidates were retrieved by individual input of gene names for AKT1, AKT2, and AKT3, as well as AKT effectors. Subsequently, they were applied to a literature survey by Google Scholar and PubMed to check their potential cell functions.

To further validate the relationship between AKT and lncRNAs to cell functions, more potential AKT-targeting lncRNAs are required. By choosing lncRNA databases, such as LncTarD [38], more AKT-targeting lncRNA candidates are generated. LncTarD is a comprehensive lncRNA database, including disease-associated lncRNA-target regulations with experiment supports, associations, and targets to biological functions, as well as TCGA pan-cancer data. By individual input target genes such as “AKT1, AKT2, and AKT3,” their respective predicted lncRNAs are generated and exported. LncTarD also provides the evidence description for each predicted lncRNA. However, these LncTarD-predicted lncRNAs for AKT1, AKT2, and AKT3 did not provide potential cell functions. Subsequently, these candidates were used for a literature survey (Google Scholar and PubMed) to check their possible cell functions. Finally, the connection between these AKT-targeting database lncRNA candidates and cell functions was established (Figure 2).

Since AKT1, AKT2, and AKT3 are encoded by different genes, their related modulating lncRNAs are different as well. This lncRNA target information for AKT1, AKT2, and AKT3 was retrieved from the LncTarD database [38] and summarized in Table 1). Several lncRNAs (ENST00113, MALAT1, CDKN2B-AS1, HULC, LUCAT1, AFAP1-AS1, LINC00462, LOXL1-AS1, AB073614, H19, and SPRY4-IT1) upregulate AKT1, while some lncRNAs (GAS5, RP11-708H21.4, FOXD2-AS1, and LINC00312) downregulate AKT1. LncRNA (lncRNA-p3134) upregulates AKT2, and lncRNA (FEZF1-AS1) upregulates AKT3.

Interestingly, the lncRNA targets for AKT1, AKT2, and AKT3 are not overlapping. Notably, the investigation of AKTs should be concerned with transcriptional regulation regarding their respective lncRNAs. The relationship between AKT1, AKT2, and AKT3 connecting to database lncRNAs in regulating cell functions will be explored further below.

#### 2.2.1. AKT1-, AKT2-, and AKT3-Targeting Database LncRNAs and Cell Functions

Although the respective AKT1-, AKT2-, and AKT3-targeting lncRNAs have been reported before (Table 1), the cell functions were not connected to these AKT1-, AKT2-, and AKT3-targeting database lncRNAs. Here, we summarize and integrate available information from our in-depth literature search on Google Scholar and PubMed (Figure 2) and provide novel information about the networking of the AKT1-, AKT2-, and AKT3-targeting lncRNAs and cell functions. Twelve AKT-targeting database lncRNAs are connected to cell functions, as summarized, especially for cancer cells (Table 2).

(1) AKT1-Targeting ENST00113 and Cell Functions

LncRNA ENST00113 modulating cell functions are summarized in Table 2). LncRNA ENST00113 enhances atherosclerosis development [62]. ENST00113 enhances proliferation and migration, but inhibits apoptosis of human umbilical vein endothelial cells by phosphorylating PI3K/AKT/mTOR [62]. AKT inactivation by inhibitor or siRNA suppresses atherosclerosis by upregulating the autophagy of macrophages [63]. Accordingly, this warrants a detailed assessment of the impact of ENST00113 on modulating autophagy and careful examination of cell functions due to ENST00113 on cancer cells in the future.

(2) AKT1-Targeting MALAT1 and Cell Functions

MALAT1 modulating cell functions are summarized in Table 2. MALAT1 enhances the proliferation and autophagy of glioma cells [65]. MALAT1 inhibition suppresses oxygen-glucose deprivation/reoxygenation-triggered apoptosis, and ER stress [66]. Upregulation of mitochondrial fusion protein mitofusin 1 (MFN1) reverses microvascular dysfunction and cardiac microvascular endothelial cell damage enhanced by MALAT1 knockdown by suppressing mitochondrial fission and apoptosis [64]. MALAT1 enhances proliferation and suppresses the senescence of gallbladder cancer cells [67]. MALAT1 improves the proliferation and migration of colon cancer cells [68]. This deserves a careful examination of cell functions due to MALAT1 to provide more evidence for impacts on cancer cells in the future.

(3) AKT1-Targeting GAS5 and Cell Functions

LncRNA GAS5 modulating cell functions are summarized in Table 2. GAS5 upregulation inhibits proliferation and promotes apoptosis of pituitary neuroendocrine [69] and liver [70] cancer cells. GAS5 induces autophagy of colon [71] and breast [72] cancer cells. GAS5 blocks high glucose-induced ER stress and apoptosis of retinal epithelial cells [73]. GAS5 pathways impact ferroptosis-associated gene expressions in heart-failure tissue [74]. GAS5 knockdown increases cell viability in the hypoxia-reoxygenation model by reducing the expression of necrosis markers, such as lactate dehydrogenase [75]. GAS5 upregulation suppresses DNA repair to ionizing radiation [76]. GAS5 overexpression blocks the senescence of vascular smooth muscle cells [77]. GAS5 upregulation blocks propofol-induced migration of glioma cells [78]. Accordingly, some cell functions of GAS5 were not investigated in cancer cells. Careful examination of some cell functions due to GAS5 acting on cancer cells is needed.

(4) AKT1-Targeting CDKN2B-AS1 and Cell Functions

CDKN2B-AS1 modulating cell functions are summarized in Table 2. CDKN2B-AS1 inhibits apoptosis and senescence of cervical cancer, which can be reverted by CDKN2B-AS1 silencing [79]. CDKN2B-AS1 upregulation induces autophagy of idiopathic pulmonary fibrosis [80]. Additionally, stress-associated endoplasmic reticulum protein 1 (SERP1) downregulates CDKN2B-AS1 and ER stress of oxygen deprivation-induced injury in cardiomyocytes [81]. CDKN2B-AS1 modifies senescence and apoptosis, involving cell cycle disturbance and DNA damage [82]. CDKN2B-AS1 improves the proliferation and metastasis of liver cancer cells [83]. Careful examination of cell functions and CDKN2B-AS1 is needed to provide more evidence for impacts on cancer cells in the future.

(5) AKT1-Targeting HULC and Cell Functions

HULC modulating cell functions are summarized in Table 2. HULC suppresses apoptosis of osteosarcoma [84] and lung cancer cells [85]. HULC induces autophagy of liver cancer cells [86]. HULC enhances the DNA repair of liver cancer stem cells [87]. Additionally, hepatitis B virus X protein upregulates HULC and downregulates senescence protein p18 expressions, suggesting that HULC can modulate cellular senescence [88]. HULC enhances the migration of pancreatic [89] and liver [90] cancer cells. This warrants a detailed examination of cell functions influenced by HULC to provide more evidence for their impacts on cancer cells in the future.

(6) AKT1-Targeting LUCAT1 and Cell Functions

LUCAT1 modulating cell functions are summarized in Table 2. LUCAT1 overexpression induces autophagy and metastasis, but suppresses apoptosis of lung cancer cells and promotes its cisplatin resistance [91]. LUCAT1 is reported to be the ferroptosis-related lncRNA correlated with renal cancer survival [92]. LUCAT1 is reported to be the necroptosis-related lncRNA in liver tumors [93]. LUCAT1 suppresses DNA damage and apoptosis of colon cancer cells [94]. A detailed examination of cell functions and LUCAT1 is warranted to provide more evidence for their impacts on cancer cells in the future.

(7) AKT1-Targeting RP11-708H21.4, AFAP1-AS1, LINC00462, and Cell Functions

RP11-708H21.4, AFAP1-AS1, and LINC00462 modulating cell functions are summarized in Table 2. RP11-708H21.4 has low expression in colon cancer cells. RP11-708H21.4 overexpression decreases proliferation and migration and triggers apoptosis of colon cancer cells by dephosphorylating AKT and mTOR [95]. Additionally, AFAP1-AS1 silencing decreases proliferation and migration and induces apoptosis of lung cancer cells [96]. LINC00462 silencing suppresses high glucose-triggered apoptosis of renal tubular epithelial cells [97]. LINC00462 improves the invasion of pancreatic cancer cells [98]. A detailed assessment of cell functions influenced by RP11-708H21.4, AFAP1-AS1, and LINC00462 is warranted to provide more evidence for their impacts on cancer cells in the future.

(8) AKT1-Targeting LOXL1-AS1, FOXD2-AS1, AB073614, and Cell Functions

LOXL1-AS1, FOXD2-AS1, and AB073614 modulating cell functions are summarized in Table 2. LOXL1-AS1 suppresses proliferation and migration and enhances apoptosis of breast cancer cells [99]. The lncRNA FOXD2-AS1 knockdown decreases proliferation and migration but triggers apoptosis of glioma cells [100]. AB073614 enhances proliferation and triggers apoptosis of cervical cancer cells [101]. AB073614 improves metastasis of gastric cancer cells [102]. This warrants a detailed assessment of cell functions influenced by LOXL1-AS1, FOXD2-AS1, and AB073614 to provide more evidence for their impacts on cancer cells in the future.

(9) AKT1-Targeting H19 and Cell Functions

H19 modulating cell functions are summarized in Table 2. H19 silencing blocks proliferation and triggers apoptosis of vascular smooth muscle cells [103]. H19 upregulation enhances invasion and autophagy of trophoblast cells [104]. H19 knockdown promotes resveratrol-induced ER stress and necroptosis of gastric cancer cells by increasing GRP78, receptor-interacting serine/threonine-protein kinase 1 (RIPK1), and mixed lineage kinase domain-like (MLKL) expressions [105]. H19 silencing enhances the ferroptosis of lung cancer cells [107]. Additionally, lncRNA H19 downregulates mitochondrial fusion expression of the MFN2 gene in renal tissues of diabetic rats [106]. H19 silencing suppresses ionizing radiation-induced DNA damage of lung cancer cells, but enhances DNA repair [108]. H19 triggers the senescence of cardiomyocytes [109]. Accordingly, some cell functions of H19 have not been investigated in cancer cells. Careful examination of some cell functions influenced by H19 is needed to provide more evidence for their impacts on cancer cells in the future.

(10) AKT1-Targeting SPRY4-IT1, LINC00312, and Cell Functions

SPRY4-IT1 and LINC00312 modulating cell functions are summarized in Table 2. SPRY4-IT1 downregulation improves apoptosis of pancreatic cancer cells [110]. SPRY4-IT1-expressing primary human melanocytes show gene expression changes along with apoptosis and DNA damage responses [111]. SPRY4-IT1 enhances metastasis in nasopharyngeal cancer cells [112]. LINC00312 suppresses proliferation and triggers apoptosis of lung cancer cells [113]. LINC00312 suppresses DNA repair of nasopharyngeal cancer cells by targeting DNA-PKcs [53]. LINC00312 suppresses the migration of bladder cancer cells [114]. A detailed assessment of cell functions influenced by SPRY4-IT1 and LINC00312 is warranted to provide more evidence for their impacts on cancer cells in the future.

(11) AKT2-Targeting LncRNA-p3134 and Cell Functions

lncRNA-p3134 modulating cell functions are summarized in Table 2. For AKT2, lncRNA-p3134 upregulation suppresses the β-cell apoptosis of pancreatic β-cells [115]. According to our literature survey, other cell functions related to AKT2 have not been reported.

(12) AKT3-Targeting FEZF1-AS1 and Cell Functions

FEZF1-AS1 modulating cell functions are summarized in Table 2. For AKT3, FEZF1-AS1 exhibits higher expression in ovarian cancer tissues and cells than normal controls [116]. Ovarian cancer patients with high FEZF1-AS1 show a poor prognosis. FEZF1-AS1 silencing inhibits proliferation and induces apoptosis of ovarian cancer cells [116]. Similarly, FEZF1-AS1 is overexpressed in gastric tumors. FEZF1-AS1 overexpression improves proliferation and autophagy of gastric cancer cells, reverted by ATG5 silencing [117]. A detailed assessment of cell functions influenced by FEZF1-AS1 is warranted to provide more evidence for their impacts on cancer cells in the future.

#### 2.2.2. Potential Future Directions

As described above, a literature survey connected AKT1-, AKT2-, and AKT3-targeting database lncRNAs to several cell functions. However, most information was derived from AKT1 in our survey (Table 2). AKT2 and AKT3 were rarely investigated. This warrants a detailed assessment of the role of AKT2 and AKT3 targeting by lncRNAs in regulating cancer cell functions in the future. Some lncRNAs reported in some cell functions but not others are based on a literature survey. Their possible contributions to unreported cell functions are not excluded and need further inspection.

### 2.3. Connecting AKT Effectors and LncRNAs to Cell Functions

AKT controls the expressions of several downstream effectors. In turn, AKT effectors exert comprehensive cell functions [3,9,10,11]. Since AKT had a cross-relationship to lncRNAs as described above, lncRNAs may exhibit the impact on most AKT effectors (FOXO, c-Myc, mTORC1, SREBP1, and HIF) (Table 3). The connection between lncRNAs to other AKT effectors (S6K1, S6K2, and 4EBP1) was rarely reported. In the following, we summarize evidence connecting some AKT effectors (FOXO, c-Myc, mTORC1, SREBP1, and HIF) and lncRNAs to cancer cell functions (Section 2.3.1, Section 2.3.2, Section 2.3.3, Section 2.3.4, Section 2.3.5 and Section 2.3.6).

#### 2.3.1. AKT Effector (FOXO)-Regulating LncRNAs and Cell Functions

The relationship between FOXO, lncRNA, and cell functions such as autophagy, ER stress, necroptosis, DNA damage response, and senescence were rarely reported. Other functions, such as apoptosis, ferroptosis, and migration were mentioned, as follows (Table 3).

(1) Apoptosis by FOXO-Regulating LncRNAs

Several lncRNA studies investigated apoptosis modulating effects involving FOXO (Table 3). Under energy stress, FOXO upregulates FOXO-induced lncRNA 1 (FILNC1) to suppress proliferation and induce apoptosis of renal cancer cells [118]. siRNA may induce or suppress apoptosis involving FOXO. In contrast, LINC00899 silencing downregulates FOXO expression and induces apoptosis of spinal ependymoma cells [119]. A detailed assessment of apoptosis influenced by more FOXO-regulating lncRNAs is warranted to provide more evidence for their impacts on cancer cells in the future.

(2) Ferroptosis by FOXO-Regulating LncRNAs

Several lncRNA studies investigated ferroptosis modulating effects involving FOXO (Table 3). Seventeen ferroptosis-related lncRNAs were associated with gastric cancer [120] and upregulated FOXO3. Some lncRNAs are risk for gastric cancer, such as VCAN-AS1, OVAAL, PCDH10-DT, ENSG00000240661.1, RPH3AL-AS1, ITGB1-DT, LINC02915, FLJ42969, NDST1-AS1, ENSG00000247134.5, and ENSG00000248362.1). Other lncRNAs are protective for gastric cancer, such as FAM239A, LINC01210, ENSG00000265334.1, LINC01775, ENSG00000273293.1, and ENSG00000230107.1) [120]. A detailed assessment of ferroptosis influenced by more FOXO-regulating lncRNAs is warranted to provide more evidence for their impacts on cancer cells in the future.

(3) Migration by FOXO-Regulating LncRNAs

Several lncRNA studies investigated migration-modulating effects involving FOXO (Table 3). LINC00899 knockdown inhibits FOXO expression and migration of spinal ependymoma cells [119]. Oncogene E26 transformation-specific or E-twenty-six (ETS)-related gene (ERG), an oncogenic transcription factor, upregulates LINC00920 to promote the proliferation and migration of prostate cancer cells by downregulating FOXO expression [121]. A detailed assessment of migration influenced by more FOXO-regulating lncRNAs is warranted to provide more evidence for their impacts on cancer cells in the future.

#### 2.3.2. AKT Effector (c-Myc)-Regulating LncRNAs and Cell Functions

There is little information about the relationship between c-Myc, lncRNA, and cell functions, such as mitochondrial morphogenesis. Other functions, such as apoptosis, autophagy, ER stress, ferroptosis, necroptosis, DNA damage response, senescence, and migration were mentioned as follows (Table 3).

(1) Apoptosis by c-Myc-Regulating LncRNAs

Several lncRNA studies investigated apoptosis-modulating effects involving c-Myc (Table 3). In some cases, lncRNAs may regulate c-Myc by direct targeting. Lnc-EPIC1 silencing triggers apoptosis of colon cancer cells by directly binding to c-Myc and downregulating c-Myc downstream effectors [122]. However, most of the c-Myc-regulating lncRNAs did not investigate their targeting potential. Inhibition of lncRNA MIR22HG suppresses proliferation and induces apoptosis of esophageal cancer cells via downregulating c-Myc expression [123]. LncRNA KCNQ1OT1 silencing causes apoptosis of acute myeloid leukemia by decreasing c-Myc expression [124]. LINC01503 is downregulated by c-Myc silencing to induce apoptosis of lung cancer cells [125], suggesting that c-Myc may upregulate LINC01503 to inhibit the apoptosis of lung cancer cells [125]. A detailed assessment of apoptosis influenced by more c-Myc-regulating lncRNAs is warranted to provide more evidence for their impacts on cancer cells in the future.

(2) Autophagy by c-Myc-Regulating LncRNAs

Several lncRNA studies investigated autophagy-modulating effects involving c-Myc (Table 3). LncRNA may induce or suppress autophagy connected to c-Myc. c-Myc-induced lncRNA MEG3 activates mitophagy to alleviate kidney ischemia–reperfusion injury [126]. In contrast, MIR7-3HG, an Myc-dependent lncRNA, blocks the autophagy of cervical cancer cells [127]. LncRNA NFYC-AS1 silencing activates autophagy of lung cancer cells by downregulating c-Myc [128].A detailed assessment of autophagy influenced by more c-Myc-regulating lncRNAs is warranted to provide more evidence for their impacts on cancer cells in the future.

(3) ER Stress by c-Myc-Regulating LncRNAs

Several lncRNA studies investigated ER stress-modulating effects involving c-Myc (Table 3). c-Myc improves adaptive ER stress [129]. Metformin upregulates the expressions of lncRNA MALAT1 and ER stress genes, while MALAT1 knockdown in metformin-treated breast cancer cells shows reduced phosphorylation of c-Myc [130]. Accordingly, MALAT1 is a potential upstream regulator to c-Myc for triggering ER stress. IA detailed assessment of ER stress influenced by more c-Myc-regulating lncRNAs is warranted to provide more evidence for their impacts on cancer cells in the future.

(4) Ferroptosis by c-Myc-Regulating LncRNAs

Ferroptosis-modulating effects of lncRNAs involving c-Myc were reported (Table 3). Transcription factor AP-2 gamma (TFAP2C) transcriptionally activates lncRNA PCAT1 to suppress ferroptosis of prostate cancer cells by interacting with c-Myc [131]. A detailed assessment of ferroptosis influenced by more c-Myc-regulating lncRNAs is warranted to provide more evidence for their impacts on cancer cells in the future.

(5) Necroptosis by c-Myc-Regulating LncRNAs

Necroptosis-modulating effects of lncRNAs involving c-Myc were reported (Table 3). Linc00176 is highly expressed in liver cancer cells, which is activated by c-Myc. Linc00176 knockdown promotes necroptosis of liver cancer cells [132]. Accordingly, c-Myc may modulate linc00176 expression to control necroptosis. A detailed assessment of necroptosis influenced by more c-Myc-regulating lncRNAs is warranted to provide more evidence for their impacts on cancer cells in the future.

(6) DNA Damage Response by c-Myc-Regulating LncRNAs

Several lncRNA studies investigated DNA damage response-modulating effects involving c-Myc (Table 3). LncRNA may induce or suppress DNA repair connecting to c-Myc. LncRNA PVT1 improves DNA repair and suppresses cell apoptosis of nasopharyngeal cancer cells [133]. p53 activates PVT1b to reduce c-Myc transcription and suppress carcinogenesis [134]. A detailed investigation of the interaction between PVT1b and Myc in modulating DNA repair is particularly needed here. Similarly, noncoding RNA activated by DNA damage (NORAD) knockdown in neuroblastoma cells upregulates the poly [ADP-ribose] polymerase 1 (PARP1), a DNA damage sensor for DNA repair [135]. In contrast, in gene set enrichment analysis (GSEA), head neck cancer patients with low lncRNA NEAT1 expression exhibit upregulation of c-Myc and DNA repair signaling [136]. A detailed assessment of DNA damage response influenced by more c-Myc-regulating lncRNAs is warranted to provide more evidence for their impacts on cancer cells in the future.

(7) Senescence by c-Myc-Regulating LncRNAs

Several lncRNA studies investigated senescence-modulating effects involving c-Myc (Table 3). Several lncRNA studies reported senescence-suppressing results connecting to c-Myc. LncRNA PARROT, an upstream modulator of c-Myc, is downregulated in the senescence of human mammary epithelial cells [137]. c-Myc may transcriptionally activate some lncRNAs, such as USP2-AS1, to inhibit senescence and improve the proliferation of lung cancer cells [138]. C1RL-AS1 knockdown promotes the senescence of gastric cancer cells by decreasing c-Myc expression [139]. c-Myc upregulates ovarian adenocarcinoma-amplified lncRNA (OVAAL) transcription to promote tumor growth and inhibit senescence [140]. A detailed assessment of senescence influenced by more c-Myc-regulating lncRNAs is warranted to provide more evidence for their impacts on cancer cells in the future.

(8) Migration by c-Myc-Regulating LncRNAs

Several lncRNA studies investigated migration-modulating effects involving c-Myc (Table 3). Several lncRNA studies reported migration-promoting results connecting to c-Myc. LINC00665 promotes c-Myc transcriptional activity to enhance the migration of lung cancer cells [141]. LncRNA AFAP1-AS1 [142] and MIR210HG [143] strengthen the migration of lung and gastric cancer cells by upregulating c-Myc, respectively. c-Myc can bind to the LINC01050 promoter to improve transcription of LINC01050 and enhances metastasis of gastric cancer cells [144]. A detailed assessment of migration influenced by more c-Myc-regulating lncRNAs is warranted to provide more evidence for their impacts on cancer cells in the future.

#### 2.3.3. AKT Effector (mTORC1)-Regulating LncRNAs and Cell Functions

There is little information about the relationship between mTORC1, lncRNA, and cell functions. Other functions, such as apoptosis, autophagy, and migration, were mentioned as follows (Table 3).

(1) Apoptosis by mTORC1-Regulating LncRNAs

There are studies on apoptosis modulating the effects of lncRNA involving mTORC1 (Table 3). LncRNA H19 suppresses mTORC1 expression of pituitary tumors [145]. Additionally, the apoptosis-promoting effects of lncRNA were reported. LINC00998 enhances mTORC2 decay and apoptosis to suppress carcinogenesis, reverted by mTORC2 overexpression [146]. A detailed assessment of apoptosis influenced by more mTORC1-regulating lncRNAs is warranted to provide more evidence for their impacts on cancer cells in the future.

(2) Autophagy by mTORC1-Regulating LncRNAs

Several lncRNA studies investigated autophagy-modulating effects involving mTORC1 (Table 3). Autophagy-inducing or -suppressing lncRNAs connecting to mTORC1 were reported. LncRNA ZNNT1 promotes autophagy of uveal melanoma cells by mTORC1 inhibitor [147]. In contrast, HAGLR opposite strand lncRNA (HAGLROS) binds to mTORC1 components and activates mTORC1 signaling by mTOR phosphorylation to inhibit autophagy, contributing to gastric carcinogenesis [148]. A detailed assessment of autophagy influenced by more mTORC1-regulating lncRNAs is warranted to provide more evidence for their impacts on cancer cells in the future.

(3) Senescence by mTORC1-Regulating LncRNAs

Senescence-modulating effects of lncRNAs involving mTORC1 were reported (Table 3). Senescence-promoting effects of lncRNA connecting to mTORC1 were demonstrated. In non-TGF-β-treated cells, silencing of the metastasis-associated in lung adenocarcinoma transcript 1 (MALAT1) activates mTORC1 [149], associated with cell senescence in chronic obstructive pulmonary disease (COPD). Accordingly, the senescence effects of mTORC1-regulating lncRNAs were not well investigated in cancer cells. A careful examination for senescence influenced by mTORC1-regulating lncRNAs on cancer cells is warranted.

(4) Migration by mTORC1-Regulating LncRNAs

Several lncRNA studies investigated migration-modulating effects involving mTORC1 (Table 3). In particular, migration-promoting effects of lncRNA connecting to mTORC1 were reported. RHPN1-AS1 silencing blocks the migration of nasopharyngeal cancer cells by decreasing MMP 2/9 expression [150]. LINC00958 activates the mTORC1 to promote the epithelial-mesenchymal transition (EMT) and migration of liver cancer cells [151]. A detailed assessment of migration influenced by more mTORC1-regulating lncRNAs is warranted to provide more evidence for their impacts on cancer cells in the future.

#### 2.3.4. AKT Effector (SREBP1)-Regulating LncRNAs and Cell Functions

As mentioned above, the relationship between SREBP1, lncRNA, and cell functions was rarely reported. Other functions, such as apoptosis and autophagy, were mentioned as follows (Table 3).

(1) Apoptosis by SREBP1-Regulating LncRNAs

Several lncRNA studies investigated apoptosis modulating effects involving SREBP1 (Table 3). The apoptosis-promoting and -suppressing effects of lncRNA connecting to SREBP1 were reported. SREBP1, SREBP2, and lncRNA ENST00000416361 were upregulated in coronary artery disease patients, accompanied by apoptosis. Inhibition of lncRNA ENST00000416361 downregulates SREBP1 and SREBP2 [152]. In contrast, free fatty acid triggers apoptosis of liver LO2 cells associated with downregulating AC012668. Overexpression of AC012668, a lncRNA, downregulates SREBP1 expression [153]. Accordingly, the relationship between SREBP1 and apoptosis warrants a detailed investigation, especially for cancer cells.

(2) Autophagy by SREBP1-Regulating LncRNAs

Autophagy-modulating effects of lncRNAs involving SREBP1 were reported (Table 3). HAGLROS knockdown downregulates SREBP1 and induces autophagy to reduce intrahepatic cholangiocarcinoma cell proliferation [154]. A detailed assessment of autophagy influenced by more SREBP1-regulating lncRNAs is warranted to provide more evidence for their impacts on cancer cells in the future.

#### 2.3.5. AKT Effector (HIF)-Regulating LncRNAs and Cell Functions

The role of HIF in regulating lncRNA-associated ER stress, necroptosis, DNA damage response, and senescence was rarely reported. Other functions involving HIF and lncRNA are summarized in Table 3.

(1) Apoptosis by HIF-Regulating LncRNAs

Several lncRNA studies investigated apoptosis-modulating effects involving HIF (Table 3). The apoptosis-promoting and -suppressing effects of lncRNA connecting to HIF were reported. LncRNA TSLNC8 triggers apoptosis of lung cancer cells by regulating HIF-1α (HIF1A) signaling [155]. lincRNA-p21 is a target of *p53* and *HIF1A* mRNA [156]. UVB upregulates lincRNA-p21 expression to induce apoptosis in keratinocytes [156]. It raises the possibility that lincRNA-p21 triggers apoptosis by regulating HIF1A. LncRNA nuclear factor of activated T cells (NFAT) silencing suppresses hypoxia-triggered apoptosis of cardiomyocytes by enhancing HIF1A expression [157].

In contrast, JPX overexpression inhibits apoptosis of nucleus pulposus cells by upregulating HIF1A [158]. Similarly, UCA1 overexpression blocks apoptosis of breast cancer cells by HIF1A inhibitor [159]. A detailed assessment of apoptosis influenced by more HIF-regulating lncRNAs is warranted to provide more evidence for their impacts on cancer cells in the future.

(2) Autophagy by HIF-Regulating LncRNAs

Several lncRNA studies reported autophagy-modulating effects involving HIF (Table 3). The autophagy-promoting effects of lncRNA connecting to HIF were reported. Hypoxia upregulates lncRNA-MALAT1 and induces autophagy of endometrial stromal cells by upregulating HIF1A expression [160]. Hypoxia upregulates MALAT1 to trigger autophagy of vascular endothelial cell injury by downregulating HIF1A [161]. PVT1 lncRNA knockdown suppresses autophagy by downregulating HIF1A in pancreatic cancer cells [162]. A detailed assessment of autophagy influenced by more HIF-regulating lncRNAs is warranted to provide more evidence for their impacts on cancer cells in the future.

(3) Ferroptosis by HIF-Regulating LncRNAs

Ferroptosis-modulating effects of lncRNAs involving HIF were reported (Table 3). The ferroptosis-suppressing effects of lncRNA connecting to HIF were reported. Hypoxia-upregulated HIF1A/lncRNA-PMAN suppressed ferroptosis of gastric cancer cells [163]. A detailed assessment of ferroptosis influenced by more HIF-regulating lncRNAs is warranted to provide more evidence for their impacts on cancer cells in the future.

(4) DNA Damage Response by HIF-Regulating LncRNAs

DNA repair-modulating effects of lncRNA involving HIF were reported (Table 3). The DNA repair-suppressing effects of lncRNA connecting to HIF were reported. LncRNA HITT (HIF1A inhibitor at translation level) directly interacts with ataxia-telangiectasia mutated (ATM) and suppresses homologous recombination repair in human colon cancer tissues [164]. A detailed assessment of DNA damage response influenced by more HIF-regulating lncRNAs is warranted to provide more evidence for their impacts on cancer cells in the future.

(5) Migration by HIF-Regulating LncRNAs

Several lncRNA studies investigated migration-modulating effects involving HIF (Table 3). The migration-promoting and -suppressing effects of lncRNA connecting to HIF were reported. HIF1A and HIF-2α can transcriptionally activate hypoxia-responsive lncRNA MALAT1 to enhance the migration of breast cancer cells [165]. LncRNA ZFPM2-AS1 enhances the migration of liver cancer cells by upregulating HIF1A [166]. LINC00649 enhances metastasis of breast cancer cells by increasing HIF1A stability [167]. LncRNA MIR17HG improves the migration of retinoblastoma cells by increasing HIF1A expression [168]. HIF1A upregulates TM4SF1-AS1 expression to enhance the migration of liver cancer cells [169]. LncRNA FAM83A-AS1 enhances the migration of lung cancer cells by upregulating HIF1A [170]. In contrast, lncRNA TSLNC8 suppresses migration effects on lung cancer cells by regulating HIF1A signaling [155]. A detailed assessment of migration influenced by more HIF-regulating lncRNAs is warranted to provide more evidence for their impacts on cancer cells in the future.

#### 2.3.6. Potential Future Directions

As described above, several lncRNAs were mentioned to regulate AKT effectors and, in turn, control cell functions. Overexpressing or downregulating these AKT effector-regulating lncRNAs may reverse the status of cancer cell functions to improve the anticancer effects. However, the cell function mechanism for the modulating impact of lncRNAs on AKT effectors remains unclear, particularly for assessing the potential targeting to AKT effectors by lncRNAs. More experiments are warranted to improve understanding of the connection between AKT effectors and lncRNAs regulating cancer cell functions.

### 2.4. Connecting AKT Effectors and Database LncRNAs to Cell Functions

To further validate the relationship of AKT effectors and lncRNAs to cell functions, more AKT effector-targeting lncRNAs are required. By choosing an lncRNA database such as the LncTarD database [38], more AKT effector-targeting lncRNA candidates are generated. By individual input target genes such as “FOXO, c-Myc, mTOR, RPTOR, MLST8, AKT1S1, DEPTOR, RPS6KB1, RPS6KB2, 4EBP1, SREBF1, and HIF1A,” their respective predicted lncRNAs are generated and exported. However, these LncTarD-predicted lncRNAs for AKT effectors did not provide potential cell functions. Subsequently, these candidates were used for literature searches (Google Scholar and PubMed) to establish the connection between AKT effector-targeting lncRNAs and cell functions (Figure 2).

In addition to Table 3, several database lncRNAs also target AKT effectors, but their relationships to cell functions are not reported. Some lncRNA target information related to AKT effectors was retrieved from the LncTarD database [38] and summarized in Table 4). c-Myc upregulates several lncRNAs (PVT1, HOTTIP, CRNDE, CCAT2, HNF1A-AS1, SNHG1, NEAT1H19, CERNA2, TUG1, PCAT1, LINC-ROR, FILNC1, and THORLNC) and downregulates some lncRNAs (HULC, PCAT1, lncRNA-BCAT1, and PCAT6). S6K1 upregulates several lncRNAs (HOTAIR and PCGEM1) and downregulates some lncRNAs (RP11-708H21.4). SREBP1 upregulates lncRNA (LNCARSR). HIF upregulates several lncRNAs (HOTAIR, RAB4B-EGLN2, MEG3, and RPL13AP23) and downregulates some lncRNA (CPS1-IT1, MIR31HG, and MALAT1) [38].

mTORC1 consists of mTOR, regulatory-associated protein of mTOR (raptor; RPTOR), mammalian lethal with SEC13 protein 8 (MLST8), proline-rich AKT substrate of 40 kDa (PRAS40; AKT1S1), and DEP domain-containing mTOR-interacting protein (DEPTOR). After retrieval from the LncTarD database, other AKT effectors, such as RPTOR, MLST8, AKT1S1, DEPTOR, and S6K2, targeted by lncRNAs were not available and not shown (Table 4).

Interestingly, most AKT effector-targeting lncRNAs do not overlap, but some AKT effector-targeting lncRNAs overlap. The latter holds for PVT1, which can target the AKT effectors c-Myc and mTOR (Table 4 and Table 5). HOTAIR can target the AKT effectors (mTOR, S6K1, and HIF1A). Additionally, some lncRNAs may provide dual functions for different cancer cells. For example, HOTAIR is upregulated in cervical cancer cells but downregulated in oral cancer cells [38]. UCA1 is upregulated in bladder cancer cells but downregulated in colon cancer cells. Although the respective lncRNAs of these AKT effectors were reported, the cell functions were not connected to these AKT effector-associated lncRNAs.

Here, we summarize the literature search (Google Scholar and PubMed) and provide novel information for networking these AKT effector-associated lncRNAs and cell functions (Table 5) (Section 2.4.1, Section 2.4.2, Section 2.4.3, Section 2.4.4, Section 2.4.5 and Section 2.4.6). AKT effectors such as c-Myc, mTOR, S6K1, SREBP1, and HIF were included. c-Myc was the target for lncRNAs (PVT1, HOTTIP, CRNDE, HULC, CCAT2, HNF1A-AS1, PCAT1, SNHG1, lncRNA-BCAT1, NEAT1, H19, CERNA2, PCAT6, TUG1, LINC-ROR, FILNC1, and THORLNC). Their respective cell functions are listed in Table 5. Some AKT effector-targeting lncRNAs (Table 5), such as HULC, H19, MALAT1, ENST00113, RP11-708H21.4, GAS5, and lncRNA-p3134, are not described here because they are the same as AKT-targeting lncRNAs (Table 2). Therefore, detailed information on cell functions for lncRNAs targeting mTOR, S6K1, SREBP1, and HIF are shown, especially for cancer cells (Table 5).

#### 2.4.1. AKT Effector (c-Myc)-Targeting LncRNAs and Cell Functions

Several c-Myc-targeting lncRNAs and their respective cell functions (Table 5) were mentioned in detail, as follows.

(1) c-Myc-Targeting PVT1 and Cell Functions

PVT1 modulating cell functions are summarized in Table 5. PVT1 inhibits apoptosis of colon [171] and thyroid [172] cancer cells. PVT1 promotes the autophagy of liver cancer cells [173]. PVT1 upregulation suppresses inflammation-induced mitochondrial fission and enhances mitochondrial fusion of myoblasts [252]. PVT1 downregulation promotes the ferroptosis of live cancer cells [174]. Additionally, PVT1 was reported as a necroptosis-associated lncRNA of gastric cancer [175]. PVT1b, the p53-dependent PVT1 isoform, is a modulator of senescence [176]. PVT1 silencing triggers apoptosis and suppresses the radioresistance of nasopharyngeal cancer cells by inhibiting DNA repair [133]. PVT1 promotes the invasion of bladder cancer cells [177]. A careful examination of some cell functions influenced by c-Myc-targeting PVT1 is warranted to provide more evidence for their impacts on cancer cells in the future.

(2) c-Myc-Targeting HOTTIP and Cell Functions

HOTTIP modulating cell functions are summarized in Table 5. HOTTIP silencing triggers apoptosis of human retinoblastoma cells, while HOTTIP overexpression suppresses apoptosis [178]. HOTTIP knockdown suppresses proliferation and migration but causes autophagy of renal cancer cells, reverted by autophagy inhibitor [179]. HOTTIP promotes DNA repair of UV-irradiated spermatogenic cells by upregulating γH2AX and p53 expression [180]. HOTTIP is involved in regulating senescence [181]. HOTTIP enhances the proliferation and migration of osteosarcoma cells [182]. A careful examination of some cell functions influenced by c-Myc-targeting HOTTIP is warranted to provide more evidence for their impacts on cancer cells in the future.

(3) c-Myc-Targeting CRNDE and Cell Functions

CRNDE modulating cell functions are summarized in Table 5. CRNDE knockdown enhances apoptosis of colon cancer cells [183]. CRNDE enhances ATG4B-dependent autophagy of liver cancer cells [184]. Additionally, CRNDE silencing suppresses ER stress and the migration of endothelial cells [185]. Inhibition of CRNDE with oxaliplatin treatment enhances DNA damage and apoptosis of colon cancer cells, reverted by upregulating CRNDE with OXA oxaliplatin [186]. A careful examination of some cell functions influenced by c-Myc-targeting CRNDE is warranted to provide more evidence for their impacts on cancer cells in the future.

(4) c-Myc-Targeting CCAT2 and HNF1A-AS1 and Cell Functions

CCAT2 and HNF1A-AS1 modulating cell functions are summarized in Table 5. CCAT2 inhibits apoptosis of colorectal cancer cells [187]. CCAT2 induces autophagy and migration of liver cancer cells [188]. HNF1A-AS1 inhibits apoptosis of bladder cancer cells [189]. HNF1A-AS1 promotes the autophagy of liver cancer cells [190]. HNF1A-AS1 enhances the invasion of lung cancer cells [191]. A careful examination of some cell functions influenced by c-Myc-targeting CCAT2 and HNF1A-AS1 is warranted to provide more evidence for their impacts on cancer cells in the future.

(5) c-Myc-Targeting PCAT1 and Cell Functions

PCAT1 modulating cell functions are summarized in Table 5. PCAT1 knockdown triggers apoptosis of head and neck cancer cells [192]. Transcription factor AP-2 gamma (TFAP2C)-dependent PCAT1 suppresses ferroptosis of prostate cancer cells [131]. PCAT1 silencing promotes radiation-induced DNA damage [193]. PCAT1 improves the migration of laryngeal cancer cells [194]. A careful examination of some cell functions influenced by c-Myc-targeting PCAT1 is warranted to provide more evidence for their impacts on cancer cells in the future.

(6) c-Myc-Targeting SNHG1 and LncRNA-BCAT1 and Cell Functions

SNHG1 and lncRNA-BCAT1 modulating cell functions are summarized in Table 5. SNHG1 silencing triggers apoptosis and blocks the migration of liver cancer cells [195]. SNHG1 induces autophagy and invasion of bladder cancer cells [196]. Downregulation of nonsense-mediated mRNA decay (NMD) effectors (SMG1 and SMG7) upregulate SNHG1 gene expression during ER stress [197]. lncRNA-BCAT1 upregulation decreases the proliferation and invasion of colon cancer cells [198]. A careful examination of some cell functions influenced by c-Myc-targeting SNHG1 and lncRNA-BCAT1 is warranted to provide more evidence for their impacts on cancer cells in the future.

(7) c-Myc-Targeting NEAT1 and Cell Functions

NEAT1 modulating cell functions are summarized in Table 5. NEAT1 inhibits proliferation and migration and induces apoptosis of cervical cancer cells [199]. NEAT1 promotes autophagy of liver cancer cells to induce radioresistance [253]. NEAT1 overexpression inhibits ER stress and migration and promotes apoptosis in gastric cancer cells [200]. Additionally, NEAT1 blocks the homologous recombination of the DNA repair pathway to inhibit the proliferation of multiple myeloma [202]. NEAT1 inhibits the doxorubicin-triggered senescence of cardiomyocytes [203]. Exosome-derived NEAT1 enhances ferroptosis to promote sepsis-induced encephalopathy [201]. A careful examination of some cell functions influenced by c-Myc-targeting NEAT1 is warranted to provide more evidence for their impacts on cancer cells in the future.

(8) c-Myc-Targeting CERNA2 and PCAT6 and Cell Functions

CERNA2 and PCAT6 modulating cell functions are summarized in Table 5. CERNA2 downregulation suppresses proliferation and triggers apoptosis of gastric cancer cells [204]. CERNA2 silencing suppresses the migration of cervical cancer cells [205]. PCAT6 suppresses apoptosis of colon cancer cells [206]. Additionally, PCAT6 induces autophagy and improves the malignancy of colon cancer cells [207]. PCAT6 was reported to be a ferroptosis-associated lncRNA for diagnosing liver cancer cells [208]. High PCAT6 levels were linked to the worse overall survival of colon cancer, accompanied by changing base excision repair and senescence [209]. PCAT6 silencing blocks the proliferation and invasion of lung cancer cells [210]. A careful examination of some cell functions influenced by c-Myc-targeting CERNA2 and PCAT6 is warranted to provide more evidence for their impacts on cancer cells in the future.

(9) c-Myc-Targeting TUG1 and Cell Functions

TUG1 modulating cell functions are summarized in Table 5. TUG1 suppresses apoptosis of cervical cancer cells [211]. TUG1 suppresses ER stress and apoptosis of renal tubular epithelial cells [213]. TUG1 inhibits ferroptosis of hypoxia/reoxygenation treated proximal tubular epithelial cells [214]. Additionally, TUG1 silencing suppresses bupivacaine-induced DNA damage for neurotoxicity [215]. TUG1 upregulation improves the senescence of lung cancer cells [216]. TUG1 improves the autophagy of colorectal cancer cells to enhance cisplatin resistance [212]. TUG1 enhances the proliferation and invasion of osteosarcoma cells [217]. A careful examination of some cell functions influenced by c-Myc-targeting TUG1 is warranted to provide more evidence for their impacts on cancer cells in the future.

(10) c-Myc-Targeting LINC-ROR and FILNC1 and Cell Functions

LINC-ROR and FILNC1 modulating cell functions are summarized in Table 5. Breast cancer cells highly express LINC-ROR, suppressing gemcitabine-induced autophagy and apoptosis [218]. Arsenite enhances LINC-ROR expression involved in DNA repair [219]. LINC-ROR enhances the migration of pancreatic cancer cells [220]. FILNC1 knockdown suppresses apoptosis of renal cancer cells [118]. A careful examination of some cell functions influenced by c-Myc-targeting LINC-ROR and FILNC1 is warranted to provide more evidence for their impacts on cancer cells in the future.

#### 2.4.2. AKT Effector (mTOR)-Targeting LncRNAs and Cell Functions

Several mTOR-targeting lncRNAs and their respective cell functions (Table 5) were mentioned in detail.

(1) mTOR-Targeting HOTAIR and Cell Functions

HOTAIR modulating cell functions are summarized in Table 5. Propofol suppresses HOTAIR to trigger apoptosis of cervical cancer cells [221]. HOTAIR promotes the autophagy of gastrointestinal stromal cancer cells to enhance their resistance to imatinib [222]. HOTAIR upregulation suppresses the paeonol-inhibiting ferroptosis of neuronal cells [224]. Additionally, DNA damage promotes HOTAIR expression in ovarian cancer cells. HOTAIR overexpression enhances DNA damage response [225]. HOTAIR improves interleukin 6 secretion after DNA damage associated with senescence [254]. Furthermore, HOTAIR knockdown inhibits autophagy and migration of cervical cancer cells [223]. A careful examination of some cell functions influenced by mTOR-targeting HOTAIR is warranted to provide more evidence for their impacts on cancer cells in the future.

(2) mTOR-Targeting UCA1 and Cell Functions

UCA1 modulating cell functions are summarized in Table 5. Curcumin suppresses proliferation and promotes apoptosis of lung cancer cells by inhibiting UCA1 [226]. UCA1 induces autophagy of leukemia cells [227]. UCA1 inhibits ER stress to suppress ischemia/reperfusion-triggered apoptosis of cardiomyocytes [228]. UCA1 silencing upregulates dynamin-related protein 1 (DRP1) and FIS1 expression leading to mitochondria fission of pancreatic cancer cells [229]. Additionally, UCA1 silencing promotes temozolomide-induced apoptosis and DNA damage to glioma cells [230]. Coactivators of activator protein 1 (AP1) and estrogen receptor α (CAPERα) cooperate with UCA1 to induce senescence of human foreskin fibroblasts [231]. UCA1 knockdown suppresses EMT expression and migration of pulmonary fibrosis [232]. A careful examination of some cell functions influenced by mTOR-targeting UCA1 is warranted to provide more evidence for their impacts on cancer cells in the future.

#### 2.4.3. AKT Effector (S6K1/2)-Targeting LncRNAs and Cell Functions

Several S6K1/2-targeting lncRNAs, such as RP11-708H21.4 and PCGEM1, and their respective cell functions (Table 5) were mentioned in detail. RP11-708H21.4 upregulation decreases proliferation and migration and induces apoptosis of colon cancer cells [95]. Additionally, exosomal PCGEM1 enhances interleukin-1β-induced apoptosis of chondrocytes [233]. LV3-shRNA-PCGEM1 promotes baicalein-induced autophagy of prostate cancer cells [234]. PCGEM1 enhances the proliferation and migration of cervical cancer cells [235]. A careful examination of some cell functions influenced by S6K1/2-targeting RP11-708H21.4 and PCGEM1 is warranted to provide more evidence for their impacts on cancer cells in the future.

#### 2.4.4. AKT Effector (SREBP1)-Targeting LncRNAs and Cell Functions

The SREBP1-targeting lncRNA LNCARSR and its respective cell functions (Table 5) were mentioned in detail. LNCARSR silencing triggers apoptosis of osteosarcoma cells [236]. LNCARSR improves the proliferation and invasion of ovarian cancer cells [237]. A careful examination of some cell functions influenced by SREBP1-targeting LNCARSR is warranted to provide more evidence for their impacts on cancer cells in the future.

#### 2.4.5. AKT Effector (HIF)-Targeting LncRNAs and Cell Functions

Several HIF-targeting lncRNAs and their respective cell functions (Table 5) were mentioned in detail.

(1) HIF1A-Targeting CPS1-IT1 and Cell Functions

CPS1-IT1 modulating cell functions are summarized in Table 5. CPS1-IT1 overexpression triggers apoptosis of colon cancer cells, reverted by CPS1-IT1 silencing [238]. LncRNA CPS1-IT1 inhibits EMT and migration of colon cancer cells by downregulating hypoxia-induced autophagy [239]. CPS1-IT1 overexpression inhibits the proliferation and migration of glioma cells [240]. A careful examination of some cell functions influenced by HIF-targeting CPS1-IT1 is warranted to provide more evidence for their impacts on cancer cells in the future.

(2) HIF1A-Targeting MIR31HG and Cell Functions

MIR31HG modulating cell functions are summarized in Table 5. MIR31HG improves proliferation and suppresses head and neck cancer cell apoptosis [241]. Additionally, MIR31HG silencing improves the senescence phenotype of fibroblasts [242]. MIR31HG silencing suppresses the migration of neuroblastoma cells [243]. A careful examination of some cell functions influenced by HIF1A-targeting MIR31HG is warranted to provide more evidence for their impacts on cancer cells in the future.

(3) HIF1A-Targeting MEG3 and Cell Functions

MEG3 modulating cell functions are summarized in Table 5. MEG3 upregulation promotes ER stress-associated protein expressions and triggers apoptosis of esophageal cancer cells [244]. MEG3 overexpression triggers autophagy of ovarian cancer cells [245]. MEG3 silencing inhibits DRP1 expression and mitochondrial fission of podocytes, reverted by MEG3 overexpression [246]. Additionally, MEG3 silencing suppresses the ferroptosis of rat brain microvascular endothelial cells [247]. MEG3 triggers necroptosis of neuron cells [248]. MEG3 maintains endothelial function by modulating the DNA damage response [249]. MEG3 suppresses the senescence of vascular endothelial cells [250]. MEG3 decreases the proliferation and invasion of colon cancer cells [251]. A careful examination of more cell functions influenced by HIF1A-targeting MEG3 on cancer cells is warranted.

#### 2.4.6. Relationship between AKT- and AKT Effector-Targeting LncRNAs

Some AKT- and AKT effector-targeting lncRNAs do not overlap, but some overlap (Table 2 and Table 4). ENST00113 and GAS5 target AKT1 and mTOR. MALAT1 targets AKT1, mTOR, and HIFA. HULC and H19 target AKT1, c-Myc, and mTOR. RP11-708H21.4 targets AKT1, mTOR, and S6K1. LncRNA-p3134 can target AKT2 and mTOR. These results provide indirect evidence that these lncRNAs may modulate AKT to regulate some AKT effectors, such as mTOR, c-Myc, and S6K1.

Some studies provide direct evidence that these lncRNAs may modulate AKT to regulate some AKT effectors. AKT and mTOR induce macrophage autophagy, as evidenced by their inhibitors [63]. ENST00113 silencing blocks the migration of HUVEC cells, accompanied by dephosphorylation of AKT and mTOR [62]. H19 upregulation improves AKT and mTOR phosphorylation to induce invasion and autophagy of trophoblast cells, reverted by H19 knockdown [104]. Consequently, some lncRNAs can modulate AKT and AKT effectors to regulate cell functions, as shown in Figure 1.

**Figure 3:**
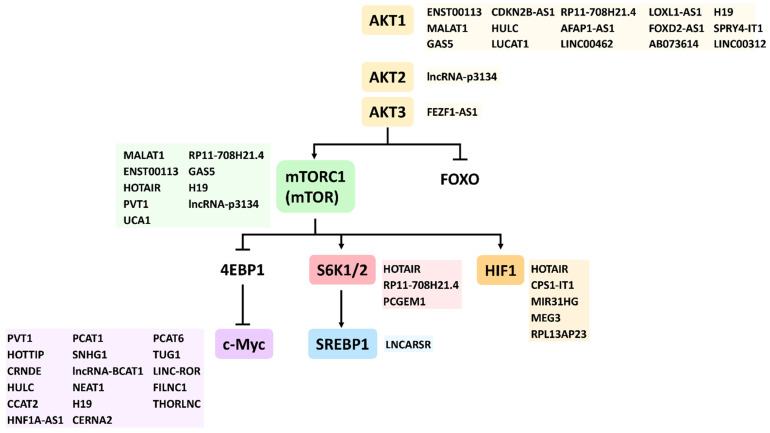
Schematic overview. AKT, its effectors, and all database lncRNAs mentioned were shown at the points of the AKT pathway that they were involved in, as shown in Table 1 and Table 4. No FOXO-targeting lncRNA was available by LncTarD searching (13 June 2022).

As described above, we provided comprehensive information for connecting AKT/AKT effectors with lncRNAs regulating cell functions. AKT1, AKT2, and AKT3 can control several AKT effectors. AKT stimulates mTORC1 through mTOR phosphorylation [255] and, in turn, suppresses 4EBP1 expression, a c-Myc negative regulator [256]. mTOR also phosphorylates and activates S6K1/2 [255] to upregulate SREBP1 expression [4]. mTOR upregulates HIF1A expression. Meanwhile, AKT inhibits FOXO expression [4,257]. However, the information for AKT- and its effector-targeting lncRNAs were arranged in different sections and tables, lacking a schematic summary. Therefore, we provide a schematic overview (Figure 3), including the AKT, its effectors, and all database lncRNAs mentioned, and show the points of the AKT pathway that they are involved in.

#### 2.4.7. Potential Future Directions

As described above, a literature survey connected AKT effector-targeting database lncRNAs to several cell functions. A careful inspection is still needed before performing more experiments to validate the targeting because they are the predicted candidates. It A deeper assessment for exploring the role of AKT effectors targeted by lncRNAs in regulating cancer cell functions is warranted.

## 3. Connecting AKT/AKT Effectors and CircRNAs to Cell Functions

In the following, the literature survey evidence to connect AKT and circRNAs to AKT signal-modulating cell functions (Figure 1) is described later (Section 3.1).

Notably, some circRNAs were reported to modulate the expressions of AKT1 [258], AKT2 [259], and AKT3 [260]. However, their potential mechanisms still warrant a detailed exploration, particularly for the possible targeting to AKT by circRNAs. Subsequently, the potential targeting to AKT1, AKT2, and AKT3 by circRNAs and their associated cell functions are discussed in Section 3.2. By choosing the circRNA database (circBase [61]), the target information of respective circRNAs was predicted, and their impacts on cell functions were evaluated, as described later.

Some circRNAs were reported to modulate the expressions of AKT effectors [61]. However, their connection to cell functions has never been investigated, especially for cancer cells. Hence, the evidence that connects the AKT effectors and circRNAs to their modulating cell functions (Figure 1) was evaluated by literature retrieval (Section 3.3). However, their potential mechanisms still warrant a detailed exploration, particularly for the possible targeting to AKT effectors by circRNAs. Subsequently, the potential targeting to AKT effectors by circRNAs and their associated cell functions are discussed in Section 3.4. By choosing the circRNA database (circBase [61]), the target information of respective circRNAs was predicted, and their impacts on cell functions were evaluated, as described later.

### 3.1. Connecting AKT and CircRNAs to Cell Functions

AKT signaling and circRNAs have a cross-relationship regulating carcinogenesis [261,262,263]. AKT-regulating circRNAs are essential in controlling apoptosis, autophagy, ER stress, senescence, and migration. The connections between mitochondrial morphogenesis, ferroptosis, necroptosis, DNA damage response, and circRNAs are rarely reported. Therefore, these cell functions with regard to apoptosis, autophagy, ER stress, senescence, and migration connecting to circRNAs are described in Section 3.1.1, Section 3.1.2, Section 3.1.3, Section 3.1.4, Section 3.1.5 and Section 3.1.6, especially for cancer cells.

#### 3.1.1. Apoptosis by AKT-Regulating CircRNAs

Apoptosis-modulating effects of circRNAs involving AKT have been reported. Several AKT-regulating circRNAs can regulate the apoptosis of cancer cells. Circ_AKT3 knockdown induces apoptosis of gastric cancer cells [264]. CircPIP5K1A overexpression suppresses apoptosis of glioma cells by phosphorylating PI3K/AKT [265]. Baicalein causes apoptosis and upregulates circHIAT1 of cervical cancer cells by dephosphorylating AKT/mTOR [266]. CircRNA_100395 overexpression induces apoptosis of gastric cancer cells by downregulating PI3K/AKT [267]. CircHIPK3 overexpression suppresses apoptosis of oral cancer cells [268]. CircRNA_0001400 knockdown triggers apoptosis of cervical cancer cells [269]. Similarly, AKT-regulating circRNAs also regulate apoptosis of noncancerous cells. CircRNA_0040414 knockdown blocks apoptosis of cardiomyocytes by downregulating PTEN and upregulating AKT [270]. Notably, some circRNA studies reported the bifunctional effects on cell functions. Circ_PRKDC knockdown induces apoptosis and autophagy in leukemia cells via dephosphorylating PI3K/AKT/mTOR [271]. Surveying more AKT-regulating circRNAs that regulate apoptosis is warranted.

#### 3.1.2. Autophagy by AKT-Regulating CircRNAs

Some circRNAs promoting and suppressing autophagy involving AKT have been reported. Several AKT-regulating circRNAs regulate the autophagy of cancer cells. CircCDR1as induces autophagy of oral cancer cells via phosphorylating AKT and ERK1/2 [272]. RNA-binding protein FUS, overexpressed in pancreatic cancer cells, upregulates circRHOBTB3 and induces autophagy by dephosphorylating AKT [273]. Similarly, AKT-regulating circRNAs also regulate the autophagy of noncancerous cells. CircRNA_103124 overexpression in Crohn’s disease triggers autophagy by dephosphorylating AKT2 [274]. In contrast, ciRS-7 suppresses starvation-triggered autophagy of esophageal cancer cells by phosphorylating AKT [275]. CircPARD3 suppresses the autophagy of laryngeal cancer cells by phosphorylating AKT [276]. Surveying more AKT-regulating circRNAs that regulate autophagy is warranted.

#### 3.1.3. ER Stress by AKT-Regulating CircRNAs

ER stress-modulating effects of circRNAs involving AKT have been reported in cancer cells. Overexpression of circCDR1as causes ER stress of oral cancer cells under a hypoxic microenvironment, accompanied by phosphorylating AKT [272]. A careful examination of ER stress influenced by more AKT-regulating circRNAs on cancer cells is warranted.

#### 3.1.4. Senescence by AKT-Regulating CircRNAs

Senescence-modulating effects of circRNAs involving AKT have been reported. AKT-regulating circRNAs may regulate the senescence of noncancerous cells. Circ_FOXO3 overexpression induces cardiac senescence, and circ_FOXO3 knockdown suppresses senescence of mouse embryonic fibroblasts [277]. By contrast, the role of AKT-regulating circRNAs in regulating the senescence of cancer cells was rarely reported. Notably, AKT phosphorylating downregulates FOXO3 during cancer development [278], and therefore the role of FOXO3 in the regulation of senescence involving AKT-regulating circ_FOXO3 needs to be examined in cancer cells. This warrants a detailed assessment of senescence influenced by more AKT-regulating circRNAs on cancer cells in the future.

#### 3.1.5. Migration by AKT-Regulating CircRNAs

Migration-modulating effects of circRNAs involving AKT have been reported in cancer cells. The migration-promoting and -suppressing effects of circRNAs connecting to AKT have been investigated. S100A4 promotes the migration of esophageal cancer cells by phosphorylating AKT [279]. ZNF139/circZNF139 enhances the migration of bladder cancer cells by phosphorylating AKT [280]. Circ_0010882 stimulates the migration of gastric cancer cells by phosphorylating AKT [261]. Similarly, circ_0002984 promotes the migration of vascular smooth muscle cells by phosphorylating AKT [281]. In contrast, circ_100395 overexpression reduced the migration of papillary thyroid cancer cells by dephosphorylating AKT [282]. This warrants surveying more AKT-regulating circRNAs that regulate migration in the future.

#### 3.1.6. Potential Future Directions

As described above, several circRNAs were mentioned to regulate AKT phosphorylation or dephosphorylation for its activation and inactivation, thereby regulating cell functions. Overexpressing or downregulating these AKT-regulating circRNAs may reverse the status of cancer cell functions to improve the anticancer effects.

However, the cell function mechanism for the modulating effects of circRNAs on AKT remains unclear, particularly for the assessment of the potential targeting to AKT by circRNAs. More experiments are warranted to improve the connection between AKT-circRNAs regulating cancer cell functions.

### 3.2. Connecting AKT1/AKT2/AKT3 and Database CircRNAs to Cell Functions

Similarly to the database for lncRNA strategy (Figure 2), AKT-targeting circRNAs were retrieved from circBase [61] by individual input target genes, such as AKT1, AKT2, and AKT3, and processed through literature search by Google Scholar and PubMed to connect their respective cell functions. Since AKT1, AKT2, and AKT3 are encoded by different genes, their related modulating circRNAs are different (Table 6). The human circRNA target information for AKT1, AKT2, and AKT3 was retrieved from circBase [61].

Interestingly, the predicted human circRNA targets for AKT1, AKT2, and AKT3 are not overlapping based on circBase retrieval (Table 6). Notably, the investigation for AKT should be concerned with transcriptional regulation regarding their respective circRNAs.

Although respective circRNAs of AKT1, AKT2, and AKT3 genes were reported, the cell functions were not connected to the AKT1-, AKT2-, or AKT3-associated circRNAs in circBase [61]. Accordingly, we searched the literature via Google Scholar and PubMed and found novel information for networking the AKT1-, AKT2-, and AKT3-associated circRNAs and cell functions, especially for cancer cells.

Although many circRNAs were shown to target AKT1, AKT2, and AKT3, only some were capable of modulating cell functions (apoptosis and migration) in cancer cells based on the literature search. Several cell functions were not reported in AKT1, AKT2, and AKT3, such as autophagy, ER stress, mitochondrial morphogenesis, ferroptosis, necroptosis, DNA damage response, and senescence.

Several kinds of AKT1-, AKT2-, and AKT3-regulating circRNAs are overexpressed in many cancer cells. For example, circAKT1 (circ_0033550) is highly expressed in cervical cancer cells (Table 6) [283]. Circ_0033550 enhances cervical tumor growth. Transforming growth factor beta (TGF-β) can upregulate circ_0033550 to promote AKT1 and EMT expression in cervical cancer cells [283]. Accordingly, circAKT1 is a potential target to slow the progression of cervical cancer development.

In addition, circAKT2 (circ_0051079) is overexpressed in osteosarcoma tissues and cell lines and enhances their proliferation and metastasis (Table 6) [284]. In contrast, circAKT2 knockdown inhibits tumor growth of osteosarcoma.

Several kinds of circAKT3 show an impact on cell functions (Table 6). CircAKT3 (circ_0017252) upregulation suppresses tumor growth and metastasis of renal cancer cells by inhibiting E-cadherin degradation [285]. CircAKT3 (circ_0017247) enhances migration and invasion of melanoma cells, reverted by circ_0017247 knockdown [286]. Similarly, circ_0017247 enhances the migration of lung cancer cells by upregulating EMT [287]. CircAKT3 (circ_0000199) upregulation increases proliferation and blocks apoptosis of oral cancer cells, reverted by silencing circ_0000199 [288].

Although the database provides many AKT-targeting circRNA candidates, most of them have rarely been investigated. A careful examination of more cell functions influenced by more AKT1-, AKT2-, and AKT3-targeting circRNAs on cancer cells is warranted. Overexpressing or downregulating these AKT-regulating database circRNAs may reverse the status of cancer cell functions to modulate their anticancer effects.

### 3.3. Connecting AKT Effectors and CircRNAs to Cell Functions

Since AKT had a cross-relationship to circRNAs as described above, the circRNAs may exhibit the impact on AKT effectors. In the following, we summarize the evidence connecting AKT effectors to circRNAs. Only some circRNAs were reported to regulate some AKT effectors (c-Myc, mTORC1, and HIF), and other AKT effectors (FOXO, S6K1, S6K2, 4EBP1, and SREBP1) were not reported. According to our literature survey (Google Scholar and PubMed), only some circRNAs could modulate cell functions (apoptosis, autophagy, and migration) (Section 3.3.1, Section 3.3.2, Section 3.3.3 and Section 3.3.4), especially for cancer cells. Several cell functions were not reported in AKT effectors, such as ER stress, mitochondrial morphogenesis, ferroptosis, necroptosis, DNA damage response, and senescence, which were not listed.

#### 3.3.1. Apoptosis and Migration by AKT Effector (c-Myc)-Regulating CircRNAs

As mentioned above, the relationship between c-Myc, circRNAs, and cell functions was rarely reported, except for apoptosis and migration. Some circRNAs can regulate apoptosis of cancer cells involving c-Myc. CircPVT1 silencing enhances apoptosis of acute lymphoblastic leukemia cells by downregulating c-Myc [289]. CircRHOT1 knockdown triggers apoptosis of lung cancer cells by decreasing c-Myc expression [290].

Additionally, some circRNAs can regulate migration involving c-Myc. The migration-promoting and -suppressing effects of circRNA connecting to c-Myc were reported in cancer cells. Several kinds of c-Myc-regulating circRNAs are overexpressed in many cancer cells. Modulating circRNAs may improve the anticancer effects by suppressing migration. For example, circZFR is overexpressed in liver cancer cells [291]. CircZFR knockdown inhibits the migration of liver cancer cells by downregulating c-Myc expression [291]. Similarly, circRNA_010763 is highly expressed in lung cancer cells. CircRNA_010763 improves the invasion of lung cancer cells by upregulating c-Myc expression [292]. Circ_NOTCH1 is overexpressed in nasopharyngeal [293] and gastric [294] cancer cells. Circ_NOTCH1 silencing inhibits the migration of nasopharyngeal cancer cells, where c-Myc can bind to the NOTCH1 promoter to transcriptionally activate circ_NOTCH1 [293]. Similarly, circ_NOTCH1 enhances metastasis of gastric cancer cells [294]. CircCCDC66 silencing decreases the migration of gastric cancer cells by downregulating c-Myc expression [295]. In contrast, circCDYL overexpression by plasmids blocks the migration of bladder cancer cells by reducing c-Myc expression [296]. Surveying more c-Myc-regulating circRNAs that control apoptosis and migration is warranted.

#### 3.3.2. Apoptosis, Autophagy, and Migration by AKT Effector (mTORC1)-Regulating CircRNAs

As mentioned above, the relationship between mTORC1, circRNAs, and cell functions was rarely reported, except for apoptosis, autophagy, and migration. Several kinds of mTORC1-regulating circRNAs are overexpressed in many cancer cells. Some circRNAs can regulate apoptosis involving mTORC1. Circ_ZNF512 knockdown inhibits apoptosis to reduce myocardial tissue injury by downregulating mTORC1 [297].

Some circRNAs can regulate autophagy involving mTORC1 [297,298]. The autophagy-promoting and -suppressing effects of circRNAs connecting to mTORC1 were reported. Circ_FOXO3 enhances autophagy of brain microvascular endothelial cells by inhibiting mTORC1 [298]. In contrast, circ_ZNF512 knockdown enhances autophagy of cardiomyocytes by downregulating mTORC1 expression [297].

Some circRNAs can regulate migration involving mTORC1. LDLRAD3 silencing decreases the migration of lung cancer cells by dephosphorylating mTOR for mTORC1 inactivation [299]. A connection between migration, mTORC2, and cicrRNAs has not been published as yet. Surveying more mTORC1-regulating circRNAs that control apoptosis is warranted.

#### 3.3.3. Apoptosis and Migration by AKT Effector (HIF)-Regulating CircRNAs

Some circRNAs can regulate apoptosis involving HIF. The apoptosis-promoting and -suppressing effects of circRNA connecting to HIF were reported. CircVEGFC improves high glucose-promoted apoptosis of vascular endothelial cells by downregulating HIF1A [300]. Circ_0010729 suppresses apoptosis of vascular endothelial cells by upregulating HIF1A [301]. In contrast, circRNA_100859 is overexpressed in colon cancer tissues and suppresses apoptosis by downregulating HIF1A [302].

Some circRNAs can regulate migration involving HIF. The migration-promoting and suppressing effects of circRNA connecting to HIF were reported. Several kinds of HIF-regulating circRNAs, such as circAGFG1, circASXL1, circ-0046600, and circPVT1, are overexpressed in many cancer cells [303,304,305,306]. Modulating HIF1A can regulate the levels of certain circRNAs. For example, circAGFG1 is highly expressed in lung cancer cells. CircAGFG1 enhances the migration of lung cancer cells by upregulating HIF1A [303]. Inhibition of circASXL1 blocks migration and HIF1A expression of lung cancer cells [304]. Circ-0046600 knockdown suppresses the migration of liver cancer cells by upregulating HIF1A [305]. Overexpressed circPVT1 enhances the migration of breast cancer cells by overexpressing HIF1A [306]. In contrast, HIF1A-regulating circRNA such as circ_EPHB4 is downregulated in cancer cells. Overexpression of circ_EPHB4, exhibiting low levels in liver cancer cells, suppressed the migration by downregulating HIF1A [307]. Surveying more HIF-regulating circRNAs that control apoptosis is warranted.

#### 3.3.4. Potential Future Directions

As described above, several circRNAs were mentioned to regulate AKT effectors and, in turn, control cell functions. Overexpressing or downregulating these AKT effector-regulating circRNAs may reverse the status of cancer cell functions to improve anticancer effects.

However, the cell function mechanism for the modulating effects of circRNAs on AKT effectors remains unclear, particularly for the assessment of the potential targeting to AKT effectors by circRNAs. More experiments are warranted to improve the connection between AKT effectors and circRNAs regulating cancer cell functions.

### 3.4. Connecting AKT Effectors and Database-CircRNAs to Cell Functions

Similarly to the database lncRNA strategy (Figure 2), AKT effector-targeting circRNAs were retrieved from circBase [61]. For the input target genes FOXO, c-Myc, mTOR, RPTOR, MLST8, AKT1S1, DEPTOR, RPS6KB1, RPS6KB2, 4EBP1, SREBF1, and HIF1A, their respective predicted circRNAs were generated and exported. Subsequently, they were processed through the literature search (Google Scholar and PubMed) to connect their respective cell functions.

In addition to Section 3.3, several circRNAs also target AKT effectors, but their relationship to cell function is not reported. Some circRNA target information to AKT effectors was retrieved from circBase [61] (Table 7).

Interestingly, the AKT effector-targeting circRNAs do not overlap (Table 7). Although the respective circRNAs of these AKT effectors were reported, the cell functions were not connected to these AKT effector-associated circRNAs.

Here, we summarize the literature search on Google Scholar and PubMed, which provided novel information for networking these AKT effector-associated circRNAs and cell functions (Table 8). Several cell functions were not reported in AKT effectors, such as autophagy, ER stress, mitochondrial morphogenesis, ferroptosis, necroptosis, DNA damage response, and senescence, which were not listed (Table 8).

For example, circMYC (circ_0085533) is more expressed in melanoma tissues than in normal tissues [308]. CircMYC knockdown suppresses cell proliferation and apoptosis of melanoma cells, reverted by circMYC overexpression [308] (Table 8).

circmTOR (circ_0009805) is overexpressed in severe preeclamptic placentas [309]. CircmTOR (circ_0009792) is upregulated in the proliferation of vascular smooth muscle cells [310] (Table 8). Other cell functions were rarely reported for this circmTOR. This warrants a detailed investigation of more cell functions involving circmTOR.

circAKT1S1 (circ_0000950) is highly expressed in the cell models of Alzheimer’s disease. CircAKT1S1 induces apoptosis of neuron cells [311]. CircAKT1S1 silencing promotes proliferation and suppresses apoptosis of neurons [312] (Table 8). Accordingly, it is a potential target to slow down the progression of Alzheimer’s disease.

circHIF1A (circ_0032138) upregulation enhances proliferation and metastasis of breast cancer cells and tissue, reverted by circHIF1A silencing [313] (Table 8). CircHIF1A (circ_0006393) is downregulated in glucocorticoid-induced osteoporosis [314]. In contrast, circ_0006393 overexpression upregulates osteogenesis-associated gene expression [314].

Although the database provides many AKT-targeting circRNA candidates, most of them were rarely investigated. Only a few AKT effector-targeting circRNAs were reported to connect to some functions of cancer and noncancer cells. This warrants a detailed evaluation of more cancer cell functions in AKT effector-targeting circRNAs in the future. Overexpressing or downregulating these AKT effector-regulating database circRNAs may reverse the status of cancer cell function to improve anticancer effects.

## 4. Conclusions

Several lncRNAs and circRNAs may regulate numerous pathways and control diverse cell functions, which are not unique to AKT and AKT effectors. For the sake of their critical regulations, as mentioned above, this review focused on AKT and AKT effectors modulating by lncRNAs and circRNAs; however, the impact of lncRNAs and circRNAs on AKT and AKT effectors in modulating cell function remains unclear. This systematic review aimed to organize the current knowledge for connecting AKT and AKT effectors to lncRNAs and circRNAs. The collected literature herein suggests that these AKT-lncRNA, AKT-effector lncRNA, AKT circRNA, and AKT-effector circRNA connections are responsible for regulating several cancer cell functions.

Databases for lncRNAs and circRNAs, such as LincTarD and circBase, provide comprehensive AKT- and AKT effector-targeting candidates for lncRNAs and circRNAs. However, their impacts on cell functions were not provided in these databases. Accordingly, the potential regulation of cell functions for more AKT- and AKT effector-targeting lncRNAs and circRNAs warrants a detailed investigation. Our literature survey shows that these AKT- and AKT effector-targeting database lncRNAs and circRNAs are organized and connected to cancer cell functions. Notably, database-predicted AKT- and AKT effector-targeting lncRNAs and circRNAs may be derived from the literature on some cancer cell lines. Since the genetic profiles of different cancer cells are different, the database-predicted lncRNAs and circRNAs candidates for AKT and AKT effectors may be limited to some cancer cell types but not others. Similarly, the organized cell functions for the AKT- and AKT effector-regulating or -targeting lncRNAs and circRNAs were also reported from different cancer cells or specific environments. Careful assessment is still required where the targeting mechanisms are concerned.

Two gaps are still present in the present review. Several lncRNAs that regulate AKT and AKT effectors were surveyed and provided a reliable connection between each other. Although the emerging evidence was collected to provide updated information, these literature-survey lncRNAs still lack the potential targeting information. Another gap is the systemic update for the databases. It is possible that some new findings or data were not immediately updated in LncTarD or circBase. Hence, more experiments to provide validated information for mechanisms regulating AKT, AKT effectors, lncRNAs, and circRNAs are required in order to fill these gaps. Consequently, the validated information can provide the resource for updating the databases for lncRNAs and circRNAs.

In conclusion, this review provides relevant information for relating lncRNAs and circRNAs to AKT and its effectors in modulating several cancer cell functions. With the help of bioinformatics and a literature survey, the detailed mechanism of targeting information to AKT and AKT effectors was well connected to lncRNAs and circRNAs and organized to regulate cell functions. This work also sheds light on AKT-signaling studies investigating potential impacts on lncRNAs and circRNAs for regulating cancer cell functions.

## Figures and Tables

**Table 1:** AKT1-, AKT2-, AKT3-targeting database lncRNAs.

	AKT1		AKT2	AKT3
**Upregulate**	ENST00113 MALAT1 CDKN2B-AS1 HULC LUCAT1 AFAP1-AS1	LINC00462 LOXL1-AS1 AB073614 H19 SPRY4-IT1	lncRNA-p3134	FEZF1-AS1
**Downregulate**	GAS5 RP11-708H21.4 FOXD2-AS1 LINC00312		-	-

lncRNAs targeting several AKT effectors were retrieved from the LncTarD database [38] (accessed on 13 June 2022).

**Table 2:** Connecting AKT1-, AKT2-, and AKT3-targeting database lncRNAs to cell functions.

		Cell Functions								
	AKT1/2/3-Targeting lncRNAs	Apoptosis	Autophagy	ER Stress	Mitochondrial Morphogenesis	Ferroptosis	Necroptosis	DNA Damage Response	Senescence	Migration
AKT1	ENST00113	[62]	[63]	○	○	○	○	○	○	[62]
	MALAT1	[64]	[65]	[66]	[64]	○	○	○	[67]	[68]
	GAS5	[69,70]	[71,72]	[73]	○	[74]	[75]	[76]	[77]	[78]
	CDKN2B-AS1	[79]	[80]	[81]	○	○	○	[82]	[79,82]	[83]
	HULC	[84,85]	[86]	○	○	○	○	[87]	[88]	[89,90]
	LUCAT1	[91]	[91]	○	○	[92]	[93]	[94]	○	[91]
	RP11-708H21.4	[95]	○	○	○	○	○	○	○	[95]
	AFAP1-AS1	[96]	○	○	○	○	○	○	○	[96]
	LINC00462	[97]	○	○	○	○	○	○	○	[98]
	LOXL1-AS1	[99]	○	○	○	○	○	○	○	[99]
	FOXD2-AS1	[100]	○	○	○	○	○	○	○	[100]
	AB073614	[101]	○	○	○	○	○	○	○	[102]
	H19	[103]	[104]	[105]	[106]	[107]	[105]	[108]	[109]	[104]
	SPRY4-IT1	[110]	○	○	○	○	○	[111]	○	[112]
	LINC00312	[113]	○	○	○	○	○	[53]	○	[114]
AKT2	lncRNA-p3134	[115]	○	○	○	○	○	○	○	○
AKT3	FEZF1-AS1	[116]	[117]	○	○	○	○	○	○	○

**○** Literature could not be found on Google Scholar or PubMed (12 June 2022). The lncRNAs targeting several AKT effectors were retrieved from the LncTarD database [38] (accessed on 13 June 2022).

**Table 3:** Connecting AKT effectors to lncRNA-regulated cell functions.

Cell Functions								
Apoptosis	Autophagy	ER Stress	Mitochondrial Morphogenesis	Ferroptosis	Necroptosis	DNA Damage Response	Senescence	Migration
FOXO c-Myc mTORC1 mTORC2 SREBP1 HIF	c-Myc mTORC1 SREBP1 HIF	c-Myc	○	FOXO c-Myc HIF	c-Myc	c-Myc HIF	c-My cmTORC1	FOXO c-Myc mTORC1

Different AKT effectors may regulate various cell functions. **○** Literature could not be found by searching Google Scholar and PubMed (12 June 2022).

**Table 4:** AKT effector-targeting database lncRNAs.

	AKT Effectors						
LncRNAs	c-Myc		mTOR		S6K1	SREBP1	HIF
**Upregulation**	PVT1 HOTTIP CRNDE CCAT2 HNF1A-AS1 SNHG1 NEAT1H19	CERNA2 TUG1 PCAT1 LINC-ROR FILNC1 THORLNC	MALAT1 ENST00113 HOTAIR PVT1 H19	CRNDE HULC lncRNA-p3134 UCA1	HOTAIR PCGEM1	LNCARSR	HOTAIR RAB4B-EGLN2 MEG3 RPL13AP23
**Downregulation**	HULC PCAT1 lncRNA-BCAT1 PCAT6		UCA1 RP11-708H21.4	GAS5 HOTAIR	RP11-708H21.4		CPS1-IT1 MIR31HG MALAT1

FOXO, mTORC1 (RPTOR, MLST8, AKT1S1, and DEPTOR), S6K2, and 4EBP1 targeted by lncRNAs were omitted because they were not available after the retrieval of the LncTarD database (http://bio-bigdata.hrbmu.edu.cn/LncTarD/ or https://lnctard.bio-database.com/) [38] (accessed on 13 June 2022).

**Table 5:** Connecting AKT effectors and database lncRNAs to cell functions.

		Cell Functions								
AKT Effectors	lncRNAs	Apoptosis	Autophagy	ER Stress	Mitochondrial Morphogenesis	Ferroptosis	Necroptosis	DNA Damage Response	Senescence	Migration
**c-Myc**	PVT1	[171,172]	[173]	○	○	[174]	[175]	[133]	[176]	[177]
	HOTTIP	[178]	[179]	○	○	○	○	[180]	[181]	[179,182]
	CRNDE	[183]	[184]	[185]	○	○	○	[186]	○	[185]
	HULC	[84,85]	[86]	○	○	○	○	[87]	[88]	[89,90]
	CCAT2	[187]	[188]	○	○	○	○	○	○	[188]
	HNF1A-AS1	[189]	[190]	○	○	○	○	○	○	[191]
	PCAT1	[192]	○	○	○	[131]	○	[193]	○	[194]
	SNHG1	[195]	[196]	[197]	○	○	○	○	○	[195]
	lncRNA-BCAT1	○	○	○	○	○	○	○	○	[198]
	NEAT1	[199,200]	[199]	[200]	○	[201]	○	[202]	[203]	[199,200]
	H19	[103]	[104]	[105]	[106]	[107]	[105]	[108]	[109]	[104]
	CERNA2	[204]	○	○	○	○	○	○	○	[205]
	PCAT6	[206]	[207]	○	[208].	○	○	[209]	[209]	[210]
	TUG1	[211]	[212]	[213]	○	[214]	○	[215]	[216]	[217]
	LINC-ROR	[218]	[218]	○	○	○	○	[219]	○	[220]
	FILNC1	[118]	○	○	○	○	○	○	○	○
	THORLNC	○	○	○	○	○	○	○	○	○
**mTORC1/2**	MALAT1	[64]	[65]	[66]	[64]	○	○	○	[67]	[68]
**(mTOR)**	ENST00113	[62]	[63]	○	○	○	○	○	○	[62]
	HOTAIR	[221]	[222,223]	○	○	[224]	○	[225]	[225]	[223]
	PVT1	[171,172]	[173]	○	○	[174]	[175]	[133]	[176]	[177]
	UCA1	[226]	[227]	[228]	[229]	○	○	[230]	[231]	[232]
	RP11-708H21.4	[95]	○	○	○	○	○	○	○	[95]
	GAS5	[69,70]	[71,72]	[73]	○	[74]	[75]	[76]	[77]	[78]
	H19	[103]	[104]	[105]	[106]	[107]	[105]	[108]	[109]	[104]
	lncRNA-p3134	[115]	○	○	○	○	○	○	○	○
**S6K1/2**	HOTAIR	[221]	[222,223]	○	○	[224]	○	[225]	[225]	[223]
	RP11-708H21.4	[95]	○	○	○	○	○	○	○	[95]
	PCGEM1	[233]	[234]	○	○	○	○	○	○	[235]
**SREBP1**	LNCARSR	[236]	○	○	○	○	○	○	○	[237]
**HIF**	HOTAIR	[221]	[222,223]	○	○	[224]	○	[225]	[225]	[223]
	CPS1-IT1	[238]	[239]	○	○	○	○	○	○	[240]
	MIR31HG	[241]	○	○	○	○	○	○	[242]	[243]
	MEG3	[244]	[245]	[244]	[246]	[247]	[248]	[249]	[250]	[251]
	RPL13AP23	○	○	○	○	○	○	○	○	○

FOXO, mTORC1 complex (including RPTOR, MLST8, AKT1S1, and DEPTOR), S6K2, and 4EBP1 targeted by lncRNAs were omitted because they were not available after the retrieval of the LncTarD database [38] (13 June 2022). mTOR is one of the components of mTORC1. ○ Literature could not be found by searching Google Scholar and PubMed (12 June 2022).

**Table 6:** AKT1/AKT2/AKT3-targeting database circRNAs.

	AKT1-, AKT2-, AKT3-Targeting CircRNAs					
**AKT1**	circ_0101403	circ_0101404	circ_0033555	circ_0033560	circ_0033559	circ_0033546
	circ_0033552	circ_0033558	circ_0033557	circ_0033553	circ_0033547	circ_0033551
	circ_0033548	circ_0033556	circ_0033549	circ_0033554	**circ_0033550**	
**AKT2**	circ_0051068	circ_0051082	circ_0051074	circ_0051077	circ_0051071	circ_0051081
	circ_0051073	circ_0051080	circ_0051070	circ_0051075	circ_0051069	circ_0051072
	circ_0051078	**circ_0051079**	circ_0051076	circ_0008719	circ_0005812	
**AKT3**	circ_0017242	circ_0112774	circ_0017251	circ_0112785	circ_0017249	circ_0112773
	circ_0006696	**circ_0017252**	circ_0017243	circ_0112797	circ_0112778	circ_0112800
	circ_0004649	circ_0112777	circ_0017254	circ_0017246	circ_0112788	circ_0112798
	circ_0017250	**circ_0000199**	circ_0112770	circ_0112782	circ_0112787	**circ_0017247**
	circ_0017244	circ_0112767	circ_0112775	circ_0017253	circ_0112799	circ_0112776
	circ_0002240	circ_0112801	circ_0112802	circ_0112772	circ_0112780	circ_0112771
	circ_0112791	circ_0112766	circ_0112783	circ_0017245	circ_0112790	circ_0112786
	circ_0112792	circ_0112768	circ_0112784	**circ_0017248**	circ_0112779	circ_0112796
	circ_0112769	circ_0112789	circ_0112781	circ_0112794	circ_0112793	circ_0112795

Data were available in the circBase database (http://www.circbase.org/) (accessed on 13 June 2022). CircRNAs in bold font were reported in the literature, as described below.

**Table 7:** AKT effector-targeting database circRNAs.

AKT Effectors	AKT Effector-Targeting CircRNAs							
**FOXO**	○							
**c-Myc**	circ_0085535	**circ_0085533**	circ_0085534					
**mTOR**	circ_0110437	circ_0009803	circ_0009829	circ_0110441	circ_0009793	circ_0009810	circ_0110442	circ_0009787
	circ_0009779	circ_0009834	circ_0009801	circ_0009795	circ_0009813	circ_0009809	circ_0110447	circ_0110418
	circ_0009837	circ_0009820	circ_0009822	circ_0009831	circ_0009844	circ_0009825	circ_0009776	circ_0009835
	circ_0009845	circ_0009847	circ_0009782	circ_0009842	circ_0009788	circ_0009819	circ_0110416	circ_0110435
	circ_0009823	circ_0009832	circ_0009839	circ_0009785	circ_0009830	circ_0009840	circ_0009808	circ_0110438
	circ_0009815	circ_0009804	circ_0009799	circ_0009800	circ_0009811	circ_0009784	circ_0009778	circ_0009821
	circ_0009786	circ_0110414	circ_0110424	circ_0009789	circ_0009828	circ_0009838	circ_0009833	circ_0110440
	circ_0009780	circ_0009777	circ_0009802	circ_0009806	circ_0009796	circ_0009798	circ_0009794	circ_0009791
	circ_0110417	circ_0006576	circ_0009826	circ_0110420	circ_0110439	circ_0009797	**circ_0009805**	circ_0009790
	circ_0009846	circ_0009807	**circ_0009792**	circ_0009814	circ_0009824	circ_0009843	circ_0009818	circ_0009841
	circ_0110415	circ_0009817	circ_0009783	circ_0110443	circ_0009812	circ_0009781	circ_0110419	circ_0009827
	circ_0009816	circ_0009836						
**RPTOR**	○							
**MLST8**	circ_0105204	circ_0037498						
**AKT1S1**	circ_0051983	**circ_0000950**	circ_0051984					
**DEPTOR**	circ_0135616	circ_0085412	circ_0135615	circ_0135617				
**S6K1**	circ_0008625	circ_0044904	circ_0044907	circ_0044900	circ_0044903	circ_0107292	circ_0107290	circ_0044902
	circ_0044905	circ_0044899	circ_0044906	circ_0107291	circ_0044901			
**S6K2**	circ_0023096	circ_0023090	circ_0023094	circ_0023095	circ_0023091	circ_0023092	circ_0023093	circ_0023089
**SREBP1**	○							
**HIF**	circ_0102309	circ_0102321	circ_0102322	circ_0102317	circ_0004817	circ_0102315	circ_0004623	circ_0102323
	circ_0102313	circ_0006326	circ_0005205	circ_0032132	circ_0032139	circ_0102310	circ_0102327	circ_0032136
	circ_0102326	circ_0102311	circ_0102314	circ_0032135	circ_0102318	circ_0032137	circ_0032140	**circ_0032138**
	**circ_0007976**	circ_0032133	circ_0102325	circ_0032134	circ_0102312	circ_0102320	circ_0102316	**circ_0006393**
	circ_0102319	circ_0102324						

mTORC1 consists of the mTOR, RPTOR, MLST8, AKT1S1, and DEPTOR. Bold circRNAs were reported in the literature, as described later. **○** indicates not available in the circBase database (accessed on 13 June 2022). CircRNAs in bold were reported in the literature, as described later.

**Table 8:** Connecting AKT effectors and database circRNAs to cell functions.

		Cell Functions	
**AKT Effectors**	**circRNAs**	**Apoptosis**	**Migration**
**c-Myc**	circ_0085533 [308]	downregulate	**○**
**mTORC1/2 (mTOR)**	circ_0009805 [309], circ_0009792 [310]	**○**	**○**
**mTORC1/2 (AKT1S1)**	circ_0000950 [311,312]	upregulate	**○**
**HIF**	circ_0032138 [313], circ_0006393 [314]	**○**	upregulate

mTOR and AKT1S1 are two of the components for mTORC1. Only circRNAs for c-Myc, mTOR, AKT1S1, and HIF were available in the circBase database (accessed on 13 June 2022). **○** indicates no available after a Google Scholar and PubMed search (accessed on 13 June 2022).

## Abbreviations

AKT: AKT serine/threonine kinase; ASMCs: airway smooth muscle cells; AP1: activator protein 1; ATM: ataxia-telangiectasia mutated; GRP78 (BiP): glucose-regulated protein 78; CAPERα: estrogen receptor α; circRNAs: circular RNAs; COPD: chronic obstructive pulmonary disease; DEPTOR: DEP domain-containing mTOR-interacting protein; DNA-PKcs: DNA-dependent protein kinase, catalytic subunit; DRP1: dynamin-related protein 1; 4EBP1 (EIF4EBP1): eukaryotic translation initiation factor 4E-binding protein 1; EMT: epithelial–mesenchymal transition; ER: endoplasmic reticulum; HAGLROS: HAGLR opposite strand lncRNA; HIF: hypoxia-inducible factor; HIF1A: HIF-1α; FILNC1: FOXO-induced lncRNA 1; HITT: HIF1A inhibitor at translation level; FOXO: forkhead box transcription factors; lncRNAs: long noncoding RNAs; MALAT1: metastasis-associated in lung adenocarcinoma transcript 1; MFN1: mitofusin 1; MLKL: mixed lineage kinase domain-like; MLST8: mammalian lethal with SEC13 protein 8; mTOR (raptor, RPTOR): mechanistic target of rapamycin; mTORC1/2: mechanistic target of rapamycin complex 1/2; NFAT: nuclear factor of activated T cells; ncRNAs: noncoding RNAs; NORAD: noncoding RNA activated by DNA damage; NHEJ: nonhomologous end joining; NMD: mRNA decay; PDGF-BB: platelet-derived growth factor BB; PARP1: poly(ADP-ribose) polymerase 1; PI3K: phosphoinositide 3 kinase; PRAS40 (AKT1S1): proline-rich AKT substrate of 40 kDa; RIPK1: receptor-interacting serine/threonine-protein kinase 1; S6K1/2 (RPS6KB1/2): mTOR substrate S6 kinase 1/2; SERP1: stress-associated endoplasmic reticulum protein 1; SOX2-OT: SOX2 overlapping transcript; SREBP1 (SREBF1): sterol regulatory element-binding protein 1; TFAP2C: transcription factor AP-2 gamma; TGF-β: transforming growth factor beta.
